# Self-crowding of AMPA receptors in the excitatory postsynaptic density can effectuate anomalous receptor sub-diffusion

**DOI:** 10.1371/journal.pcbi.1005984

**Published:** 2018-02-14

**Authors:** Rahul Gupta

**Affiliations:** School of Computational and Integrative Sciences, Jawaharlal Nehru University, New Delhi, India; George Mason University, UNITED STATES

## Abstract

AMPA receptors (AMPARs) and their associations with auxiliary transmembrane proteins are bulky structures with large steric-exclusion volumes. Hence, self-crowding of AMPARs, depending on the local density, may affect their lateral diffusion in the postsynaptic membrane as well as in the highly crowded postsynaptic density (PSD) at excitatory synapses. Earlier theoretical studies considered only the roles of transmembrane obstacles and the AMPAR-binding submembranous scaffold proteins in shaping receptor diffusion within PSD. Using lattice model of diffusion, the present study investigates the additional impacts of self-crowding on the anomalousity and effective diffusion coefficient (*D*_*eff*_) of AMPAR diffusion. A recursive algorithm for avoiding false self-blocking during diffusion simulation is also proposed. The findings suggest that high density of AMPARs in the obstacle-free membrane itself engenders strongly anomalous diffusion and severe decline in *D*_*eff*_. Adding transmembrane obstacles to the membrane accentuates the anomalousity arising from self-crowding due to the reduced free diffusion space. Contrarily, enhanced AMPAR-scaffold binding, either through increase in binding strength or scaffold density or both, ameliorates the anomalousity resulting from self-crowding. However, binding has differential impacts on *D*_*eff*_ depending on the receptor density. Increase in binding causes consistent decrease in *D*_*eff*_ for low and moderate receptor density. For high density, binding increases *D*_*eff*_ as long as it reduces anomalousity associated with intense self-crowding. Given a sufficiently strong binding condition when diffusion acquires normal behavior, further increase in binding causes decrease in *D*_*eff*_. Supporting earlier experimental observations are mentioned and implications of present findings to the experimental observations on AMPAR diffusion are also drawn.

## Author summary

The transmembrane AMPA receptors (AMPARs) prominently exhibit lateral diffusion in the postsynaptic membrane at excitatory synapses. Steric obstructions to AMPAR diffusion due to the crowd of other relatively static transmembrane proteins and binding of AMPARs to the submembranous scaffold proteins in the specialized region of postsynaptic density (PSD) are well known to retard receptor diffusion, which causes receptor trapping and accumulation within PSD. However, AMPARs are significantly bulky structures and may also obstruct their own diffusion paths in the presence of their high density. It is shown here that intense self-crowding of AMPARs may lead to highly obstructed and confined receptor diffusion even in the obstacle-free medium, and the presence of other obstacles further aggravates this effect. AMPAR-scaffold binding reduces confined diffusion arising from self-crowding and strong binding engenders normal diffusion even at high receptor density. However, it overall causes reduction in the effective diffusion coefficient of the receptor diffusion.

## Introduction

Glutamate-binding transmembrane alpha-amino-3-hydroxy-5-methyl-4-isoxazole-propionic acid (AMPA)-type receptors (AMPARs) are the pivotal element of fast synaptic transmission at excitatory synapses in the central nervous system [1, 2]. At the site of synaptic contact, these receptors are present at high density within a specialized region on the postsynaptic membrane, termed as postsynaptic density (PSD), which is closely apposed to the presynaptic active zone of glutamate release [3–5]. The remaining extra-synaptic region of the postsynaptic membrane is distinguished with a relatively lower density of AMPARs [4]. This unique spatial arrangement of AMPARs is a natural adaptation to expose a sufficiently large number of the receptors to sufficiently high glutamate concentration in the synaptic cleft [6–8] and, thus, enhance the postsynaptic response, which would otherwise be comparatively weaker under a homogeneously distributed receptor condition [9–11].

Like other mobile transmembrane proteins, AMPARs also exhibit lateral diffusion in the postsynaptic membrane [12, 13]. AMPAR mobility is crucial for many essential processes associated with the efficiency of synaptic functioning. Lateral diffusion causes continuous exchange of the receptors between PSD and extra-synaptic region [14, 15]. This exchange brings about replacement of desensitized AMPARs in the PSD with active AMPARs of the extrasynaptic region after an event of glutamate release and, hence, assists in maintaining the strength of consecutive postsynaptic responses in the presence of high-frequency presynaptic spike train [16–18]. It also underlies the recruitment of new AMPARs into the PSD which are brought by exocytotic vesicles from the local intracellular reserve-pool and are initially unloaded on the extra-synaptic membrane [19, 20]. In a similar manner, the older receptors in the PSD diffuse out to the extra-synaptic region where they are endocytosed [21]. Accordingly, lateral diffusion of AMPARs assists in the appearance of long-term potentiation (LTP) [20] or long-term depression (LTD) [22, 23] at excitatory synapses and, hence, assists in the molecular basis of learning. Owing to such a crucial and indispensable role of AMPAR lateral diffusion in shaping the density and spatial localization of AMPARs in the PSD, it has always engaged attention of a wide scientific community.

A remarkable thing is the gathering of AMPARs in the PSD despite that PSD spans a much smaller area than the extra-synaptic region and the AMPARs are significantly mobile. It seems that the receptors get trapped in the PSD while diffusion because, in the absence of trapping, diffusion would lead to a homogeneous distribution of AMPARs on the entire postsynaptic membrane. In the backdrop of continuous receptor exchange between PSD and extra-synaptic region, the trapping can be viewed in terms of the considerably longer residence time [24] of an AMPAR in the PSD. Using techniques like fluorescence recovery after photobleaching (FRAP), electrophysiology with mutant variants of AMPAR, and different versions of single particle tracking such as sptPALM, uPAINT and quantum dot (QD)-tagging of receptors, various experimental studies [24–29] in hippocampal slices and live hippocampal neurons in dissociated cultures have provided a wealth of observations on the nature of AMPAR diffusion at excitatory synapses. In conjunction with the theoretical investigations [29–34], these studies have so far clearly shown that the molecular composition of the PSD is indeed responsible for the trapping of diffusing AMPARs. The PSD is rich in large number of transmembrane as well as submembrane proteins [3, 35–37], owing to which it possess a very high molecular weight [37, 38]. The crowding of inert transmembrane proteins strongly obstructs the AMPAR diffusion within the PSD region through steric repulsion [29, 31]. Further, the reversible binding of intracellular domain of AMPARs as well as their associations with transmembrane AMPAR regulatory proteins (TARPS) to the submembranous scaffold proteins, such as PSD-95, also substantially reduces the AMPARs mobility [29, 39, 40].

An AMPAR is a tetramer and is consist of any combination of the four kinds of subunits GluR1, GluR2, GluR3 and GluR4 [41, 42]. Typically, GluA1-GluA2 and GluA2-GluA3 heterotetramers are most abundant in the adult brain [43–45]. These receptors are very bulky [41] and carry along a large steric-exclusion volume. The bulkiness of AMPARs is further increased due to the various auxiliary proteins [46–50] associated with it. In fact, the size of the native complexes of AMPARs isolated through biochemical techniques have been found to be approximately double the original size of the tetramer [51]. Further, AMPARs reside at high density in the PSD and contributes to a substantial fraction of the local macromolecular crowding [3, 37]. Therefore, it is reasonable to envisage that these receptors may block the diffusion paths of each other and may lead to a situation of self-obstruction or self-crowding. Moreover, the distribution of AMPARs in the PSD is not strictly homogeneous. Rather, there are smaller subregions or nanodomains within the PSD which have more AMPARs cluttered [52, 53] and the self-crowding of these receptors would be more pronounced. Accordingly, besides inert transmembrane protein crowding and binding to scaffold proteins, self-crowding of AMPARs may appear an additional factor behind the reduced or hampered mobility and trapping of these receptors in the PSD. However, in the earlier experimental and theoretical studies, the possible role of self-crowding factor has remained completely unaddressed.

The above speculation regarding self-crowding of AMPARs is a seemingly interesting issue and, therefore, is the source of motivation for carrying out the present theoretical investigation. The effect of various crowding factors on the AMPAR diffusion can only be enquired through detailed numerical simulation of independent diffusing receptors. Therefore, the present study involves the Monte Carlo simulation of receptor diffusion using lattice model of diffusion, which has proven to be an effective approach in the earlier theoretical studies [31, 54, 55]. The main body of the present study is comprised of a purely abstract framework with a lattice used as a generalized spatially-discrete medium of diffusion, regardless of whether the lattice represents the entire PSD or a subregion within the PSD. Moreover, the AMPARs are represented by point diffusion tracers (DT) on the lattice [31, 54]. On the basis of the ensemble-averaged mean-squared displacement of tracer diffusion, the nature of diffusion is established in terms of two physical quantities viz. anomalousity and effective diffusion coefficient under the different pertinent conditions of crowding and binding events. Both the quantities serve as the suitable marker of resulting dwell time of the receptors within PSD and, hence, can be effectively used to comprehend receptor trapping [31]. It must be noted that the dynamics of the AMPAR accumulation in the excitatory PSD and exchange with extrasynaptic region is not the immediate interest of the present study. Rather, it focusses on capturing the emergent statistical behaviors of the receptor diffusion in the thermodynamic limit when self-crowding is considered in addition to the other obstacles and binding, which may be later used as the building block to comprehend the dynamics of accumulation.

The findings reveal that even in the absence of any steric crowding of other transmembrane and scaffold proteins in the postsynaptic membrane, very high density of AMPARs may itself lead to extraordinarily high anomalousity and reduced diffusion coefficient. Remarkably, anomalousity of receptor diffusion may also exhibit a switch-like behavior with respect to their self-crowding density, similar to the switch-like behavior with respect to increase in steric macromolecular crowding of other PSD proteins observed earlier [31] as well as here. Further, increase in the crowding by other PSD proteins may exacerbate the anomalousity and decline in diffusivity arising from self-crowding. Contrarily, binding appears to mark a reverse effect by decreasing the anomalousity of crowded receptor diffusion. The plausible mechanisms underlying these findings are discussed to details. Moreover, the relevance of the use of point tracers in capturing the picture of self-crowded diffusion of the non-zero lateral-sized AMPARs is also drawn. Eventually, the possible elements of self-crowding lying within the earlier experimental observations on the nature of AMPAR diffusion at excitatory synapses are pointed out and the physiological relevance of the present observations made through the abstract framework is established in regard of real biological scenario.

## Methods

### Classification of PSD macromolecular crowding into Completely-Reflecting and Partially-Reflecting-cum-Binding obstacles

**Fig 1:**
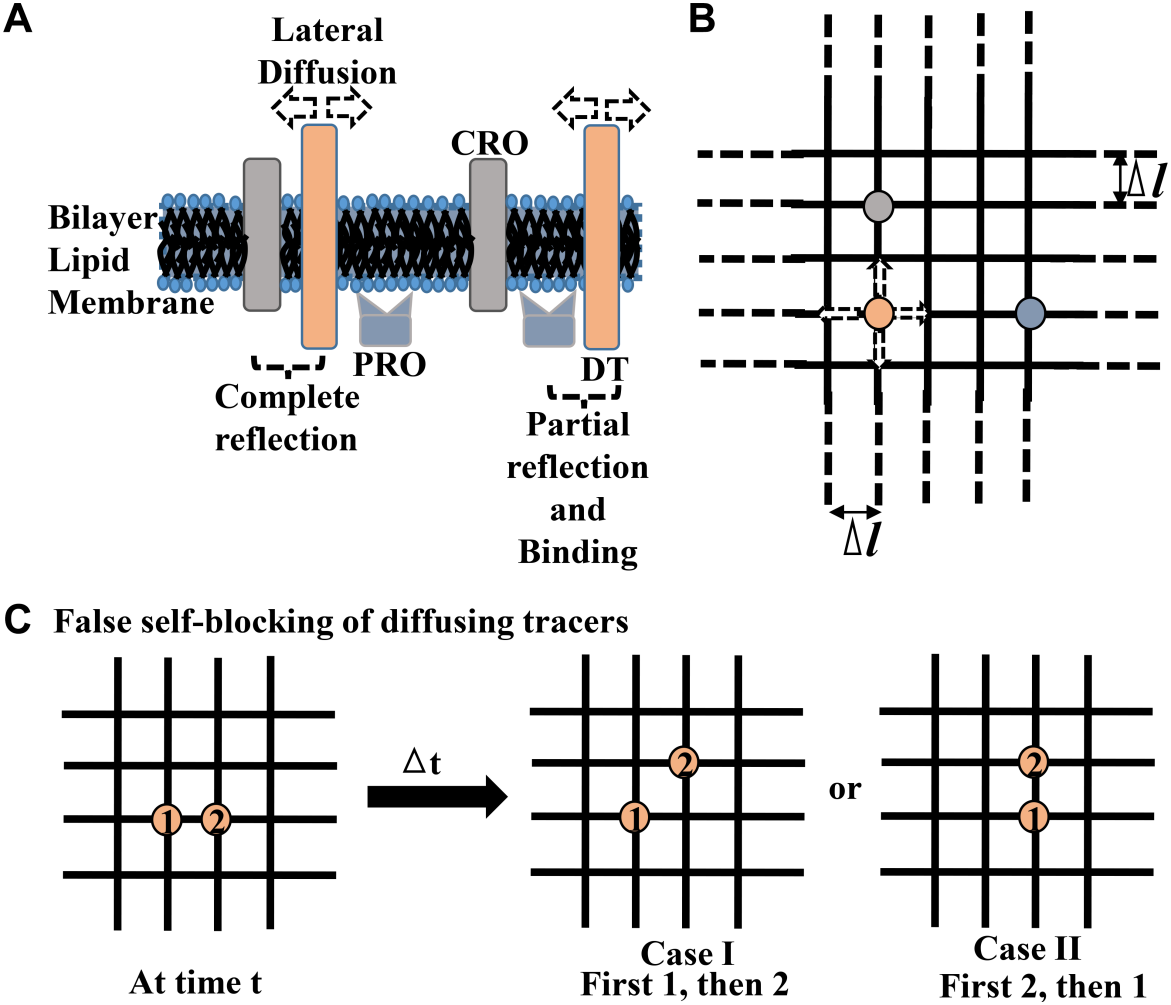
The simplified molecular composition of PSD and lattice model of AMPA receptor diffusion. (A) An schematic demonstration of the CROs and PROs under the broad classification adopted here for the various crowding elements present at the PSD of a typical excitatory synapse on a dendritic spine. CROs are generally the transmembrane proteins which interact with a diffusing receptor only to elastically repel it away on collision. PROs are typically the submembranous scaffold proteins which offer partial reflection as well as binding to diffusing receptors. The AMPAR lateral diffusion is modeled here using a transmembranous diffusing tracer (DT). (B) A part of the entire lattice illustrating a discrete space for the tracer diffusion. DT, CRO and PRO are localized on the lattice as point elements, with different respective area fractions. A tracer can diffuse randomly to either of the four directions. Δ*l* is the size of the lattice edges between any two lattice points. The entire lattice is an abstraction meant to be used here for representing receptor diffusion over either the entire PSD or a region within the PSD. (C) An illustration of the false self-blocking of two DTs, labelled 1 and 2, lying at the neighbouring lattice sites. The different computational sequences of performing the hopping of these DTs may lead to different new configurations of the tracers localizations on the lattice. One of these configurations is false, viz. Case I, with respect to the ideal concept of simultaneous diffusion of the tracers over a time-step of simulation. Its description is provided in the text.

The lateral diffusion of AMPARs is realized here through the numerical simulation of the diffusion of DTs. For simplicity, the entire macromolecular crowding at the PSD can be broadly classified into two pools [31]: Completely-Reflecting Obstacles (CROs) and Partially-Reflecting-cum-Binding Obstacles (PROs) (Fig 1A). In general, the CROs represent various transmembrane proteins in the PSD [3, 35] which interact with a diffusing AMPAR only to reflect it away on collision via. steric repulsion. Since they never significantly bind the AMPARs, they behave as inert obstacles [31, 54]. On the other hand, the PROs are often the submembranous scaffold proteins which are intracellularly accumulated close to the PSD [3, 35]. These proteins generally offer only a partial obstruction to the diffusing AMPARs through steric repulsion of the intracellular C-terminal domains of the receptors [31]. Moreover, they preferentially bind the receptors almost at their location through reversible non-covalent interactions between specific domains of the receptors and the scaffold proteins. For instance, GluR1 subunit of AMPARs can directly bind to the scaffolding SAP-97 proteins [56] whereas GluR2 subunits can bind to submembranous PICK1 or GRIP [57]. However, AMPARs cannot directly interact with the one of the most abundant PSD-95/SAP-90 scaffold proteins at excitatory synapses due to their incompatible PDZ domains. Rather, the receptors require association with auxiliary transmembrane AMPA receptor regulatory proteins (TARPs), such as Stargazin, to bind with PSD-95 [39].

Accordingly, the probability of reflection (*P*_*reflect*_) for tracer collision with CROs is always 1 [31] whereas it may be variable for collision with PROs, as it depends on the size of the C-terminal domains of AMPAR subunits, the size of the specific scaffold proteins, presence of auxiliary proteins etc. For the present investigation, *P*_*reflect*_ for collision with PROs is kept fixed at 0.5, signifying a 50% chance of reflecting the trajectory of a tracer on physical contact without binding [31]. Furthermore, the detailed multi-step kinetic scheme for the binding of AMPARs to scaffold proteins is still unknown. However, the intensity of binding through hydrogen bond interactions within PDZ domains have been estimated to be in the range of 2–13*k*_*B*_*T* [31, 58–60], which may serve here as a rough estimate for the binding energy of AMPAR-scaffold interactions.

Accordingly, three specific situations of homogeneous DT-PRO binding viz. weak, intermediate and strong bindings with binding energy 2, 6 and 10 *k*_*B*_*T*, respectively, are taken into account in the present investigation. Further, a conventional approach is to assume the entire crowding factors to be static at their spatial locations in the PSD while an AMPAR diffuses through the crowd [29, 31]. This approach is reasonably correct to a great extent as the mobility of the crowding factors is too low [61, 62] in comparison to that of AMPARs and their average life-time in the PSD (in hours) [63] is substantially high relative to the typical measurement time-duration of AMPAR diffusion (in seconds). Accordingly, the obstacles CROs/PROs are considered here immobile and their density preserved throughout the duration of tracer diffusion.

### Lattice model of diffusion

A two-dimensional square lattice (Fig 1B) is considered for performing the lateral diffusion of AMPARs in the postsynaptic membrane [31, 54, 55, 64]. The lateral diffusion coefficient of an AMPAR in the extra-synaptic membrane is known to be almost 0.2 × 10^−3^*μm*^2^.*ms*^−1^ [24, 31, 65]. Since the extrasynaptic membrane offers least macromolecular obstructions relative to the PSD, this estimate is considered here as the natural free diffusion coefficient of an AMPAR in an unobstructed lipid medium of the postsynaptic membrane and is here assigned to the effective diffusion coefficient of DT (*D*_*eff*_). The diffusion is performed at discrete time-steps Δ*t* of fixed size 10^−3^*ms* [31]. The mean-squared displacement (MSD), 〈*r*^2^〉(*t*), of a DT undergoing free normal diffusion in a two-dimensional medium is given by,

$$\begin{array}{c} \langle r^{2}\rangle (t)=4D_{eff}t \end{array}$$ (1)

Therefore, using Eq 1, the desired finite diffusion length Δ*l* for the lattice diffusion can be computed to be 8.9 × 10^−4^*μm* [31]. This estimate is assigned to the edge length between any two lattice-sites in the square lattice. In this way, the size of the entire lattice in terms of the number of lattice-sites is kept 1119 × 1119 such that the lattice approximates to an area of 1*μm*^2^ [31]. It must be noted that the lattice employed here is a purely abstract framework to procure the salient features of the tracer diffusion under different crowding conditions. Therefore, depending on the requirement, it may be used to address the properties of AMPAR diffusion over the entire PSD as well as within a subregion of the PSD.

The two kinds of obstacles, CROs and PROs, are considered as point obstacles over the lattice (Fig 1B). The CROs and PROs are uniformly distributed over the lattice according to their desired area fractions *a*_*CRO*_ and *a*_*PRO*_, respectively. The two classes of obstacles are dealt separately so that how these obstacles of different nature may affect tracer diffusion in their specific manners can be clearly examined.

Since the main objective of the present study is to investigate the effect of self-crowding of tracers on their lateral diffusion, a standard situation of “no-self-crowding” is also taken into account which serves as a benchmark for comparative analysis of the observations made under varying self-crowding situations. In this standard situation, DTs are uniformly placed on the square lattice where each tracer behaves as an independent diffusing entity. Therefore, while diffusion, two or more tracers can together occupy the same lattice site. No steric exclusion among the tracers is considered. In fact, this constitutes an ensemble of multiple copies of independently diffusing tracers but with different initial positions on the lattice under an identical distribution of CROs or PROs. While initially placing DTs on the lattice, it is taken care that a tracer should not lie at a lattice site already occupied by a CRO whereas it is allowed to lie on the lattice-site occupied by a PRO. Moreover, in the case of PROs, the tracers are allowed to diffuse for 2*s* after being initially placed to acquire thermal equilibrium. Only after this annealing period, the measurement of tracer diffusion is performed [55].

However, while dealing with the self-crowding conditions, the density of diffusing tracers placed on the square lattice would also matter (Fig 1B). Accordingly, the area-fractions of the lattice occupied by DTs, *a*_*DT*_, are taken in the increasing orders of the magnitudes such that conditions of six different *a*_*DT*_ viz. 0.00001, 0.0001, 0.001, 0.005, 0.01 and 0.1, are investigated in the present study. Here too, the DTs are uniformly distributed at the desired *a*_*DT*_ and considerations regarding their initial placement on the lattice depending on the CROs or PROs are taken care as described for the standard situation.

### Algorithm of diffusion

A periodic boundary condition is imposed on the boundary of the lattice mesh [31, 54]. Therefore, as a tracer leaves the mesh, it re-enters the mesh from the exact opposite side. Monte-Carlo simulation of tracer diffusion is performed. At each time-step, all the tracers present over the mesh are inspected for diffusion one by one. For each tracer, a random number is generated from the uniform random number distributed over the interval [0, 1] to decide the direction of its diffusion. If the random number is < 0.25, the tracer would move left. If the random number is ≥ 0.25 but < 0.5, the tracer would move right. If the random number is ≥ 0.5 but < 0.75, the tracer would move up. Finally, if the random number is ≥ 0.75, the tracer would move down. Based on this outcome, the tracer intends to hop to its nearest-neighbouring lattice site, referred to as the destination site. However, before accomplishing the hopping, the occupancy status of the destination site in regard of CRO or PRO is checked.

If the destination site is occupied by a CRO, no hopping is performed and the tracer stays at its original lattice-site. In the case of PRO occupying the destination site, a uniform random number is again generated over the interval [0, 1] to check for the partial reflection of the diffusing AMPAR with the *P*_*reflect*_ = 0.5. If the random number is ≥ *P*_*reflect*_, the tracer is allowed to diffuse to the destination site. Once the tracer reaches the PRO-occupied lattice site, it is considered to be bound. It will unbind and diffuse at a further time-step only when another uniform random number generated in a similar manner is greater than or equal to the probability of escape, *P*_*escape*_, which is defined from the binding energy as [55],

$$\begin{array}{c} P_{escape}=e^{\left(\frac{-Binding\;Energy}{k_{B}T}\right)} \end{array}$$ (2)

Once the tracer unbinds from the PRO, it is allowed to diffuse in either of the directions isotropically. Therefore, it may be noted that rotational diffusion of DT is neglected in the present framework [31].

The above algorithm is identically shared by the standard condition as well as the conditions of self-crowding. However, the latter condition involves some additional restrains for hopping to the destination site. Since steric-exclusion of DTs among themselves is present in the case of self-crowding, the destination site already occupied by another tracer does not allow hopping of the subject tracer while diffusion. Under such a situation, a peculiar phenomenon of false self-blocking of tracers might appear during diffusion simulation. This condition and its implemented remedy are described in the following subsection. Further, as long as another DT is bound to PRO, the partial reflection of the PRO turns into a complete reflection and the destination site would behave as if it is occupied by a CRO.

### Recursive algorithm for avoiding false self-blocking of DTs

While performing the lattice-diffusion of a population of DTs with steric exclusion for each other, there appears a computational problem regarding the sequence of performing the finite-step hopping of individual tracers at each time-step of the diffusion simulation. At every time-step, all the tracers are genuinely expected to diffuse simultaneously on the lattice in random directions and depending on the availability of unoccupied neighbouring sites. If the tracers do not have steric-exclusion property, more than one receptor can occupy a single lattice site. Under this assumption, the computational sequence of performing the hopping of receptors one by one during a single time-step of simulation does not matter. However, if the tracers sterically repel each other, the computational sequence of performing the hopping of tracers at each time-step may lead to different profiles of diffusion.

This issue becomes clearer when the lattice-diffusion of only two DTs, let’s say, DT1 and DT2 with steric repulsion is illustrated (Fig 1C). If the two tracers are sufficiently isolated from each other on the lattice, the sequence of performing the finite-step hopping of the individual tracers during a forward time-step of simulation does not matter, as either sequence, first DT1 and then DT2 or first DT2 and then DT1, leads to the same diffusion profile. Now consider that the two tracers are sitting at the neighbouring sites and the random number generation leads to the expected movements of DT1 towards DT2 and DT2 towards the upper unoccupied neighbouring site. The sequence where first DT1 is considered for hopping will lead to reflection of DT1 back to its position since the DT2 is presently occupying the lattice-site. Next, when DT2 is considered for hopping, it will easily move to the upper lattice site leaving behind an unoccupied lower lattice site. Here, only one receptor DT2 could practically diffuse. In another sequence where first DT2 is considered for hopping and then DT1, DT2 will move to the upper lattice site and DT1 will arrive at the earlier position of DT2. Here, both the tracers could diffuse. According to the theoretically-expected simultaneous diffusion of both the tracers, the diffusion profile deriving from the latter sequence is correct but the former sequence leads to an artefact owing to the computational sequence of performing the hopping of tracers.

To solve this problem, an algorithm with two recursive steps of performing a sequential hopping of tracers at each time-step of simulation is devised:

Step 1 At time *t*, generate uniform random numbers for all the tracers to decide their expected direction of hopping.
Step 2 Perform the regular routine check for finally hopping the tracers one by one to their destination site. Especially, the tracers whose destination neighbouring sites are occupied by the presence of a DT-associated PRO or the presence of a DT are stayed back at their original site and are labelled as “blocked by another DT”.
Step 3 On completion of step 2 for all the tracers, the set of labelled tracers are again examined for their respective previous destinations sites for the absence of DT. The labelled tracers whose destination sites are now free are diffused and the hoping of the remaining subset is rejected.
Step 4 The time is incremented to *t* + Δ*t*.

This scheme of hopping the tracers completely removes the possibility of false self-blocking of tracers while diffusion on the lattice.

### Analysis

The time-duration of the recording of spatial locations of the DTs is 2*s* [31]. Wherever necessary, the observation has been made for an extended duration of time. Using the DTs’ trajectories, the temporal profile of ensemble-averaged MSD is computed as,

$$\begin{array}{c} \langle r^{2}\rangle (t)=\frac{1}{N}\sum_{i=1}^{N}{\left(x_{i}(t)-x_{i}(0)\right)}^{2}+{\left(y_{i}(t)-y_{i}(0)\right)}^{2} \end{array}$$ (3)

Where, *N* is the number of DTs on the two dimensional lattice. *x*_*i*_ (*t*) and *y*_*i*_ (*t*) are the *x*− and *y*− coordinates of the *ith* tracer at time *t*. *x*_*i*_ (0) and *y*_*i*_ (0) denotes the initial location of the *ith* tracer at the beginning of diffusion i.e. *t* = 0. The MSD profiles are further averaged over 250-700 ensembles of lattices for every crowding conditions. In theory, the MSD of two-dimensional diffusion is described in general as

$$\begin{array}{c} \langle r^{2}\rangle (t)=4Dt^{\alpha } \end{array}$$ (4)

Here, *α* is the anomalous exponent and *D* is the diffusion constant of the diffusing particle. If *α* = 1, the diffusion is normal. However, if 0 < *α* < 1, it characterizes anomalous sub-diffusion. In this regard, computation of the log (〈*r*^2^〉(*t*)/*t*) vs log (*t*) profile, referred in the following text as log-log profile, is very beneficial for procuring many important features of the tracer diffusion.

$$\begin{array}{c} \text{log}\;(\langle r^{2}\rangle (t)/t)=\text{log}\;(4D)+(\alpha -1)\text{log}\;(t) \end{array}$$ (5)

It may be noted that for normal diffusion with *α* = 1, the log-log profile would appear a flat horizontal line with slope zero. However, for anomalous diffusion, the log-log profile would have a negative slope of magnitude (1 − *α*). Higher will be the anomalousity of diffusion, sharper will be the decline in log-log profile. Therefore, the log-log profile can easily provide a clear demarcation for the diffusion to be called normal or anomalous and is useful for computing the anomalous exponent of the diffusion as well. As a matter of fact, one may also be interested in the spatiotemporal profile i.e. probability distribution function of tracer diffusion. However, the main interest of this paper is in properties directly pertinent to tracer mobility viz. anomalousity and effective diffusion coefficient. Since a Gaussian or non-Gaussian diffusion can be normal as well as anomalous [66], the mobility factors are ultimately described by the MSD.

## Results

Lattice diffusion with no-self-crowding condition of tracers would serve as a standard benchmark for this study and corresponds to the conventional approach [29, 31, 54, 64] adopted so far in the existing literature. Accordingly, each following subsection begins with mentioning the observations made under varying conditions of reflecting obstacles (CROs) or/and binding obstacles (PROs) but in the absence of self-crowding factor. Subsequently, features of diffusion in the presence of self-crowding factor would be discussed.

### Features of tracer diffusion in the presence of completely reflecting transmembrane obstacles

**Fig 2:**
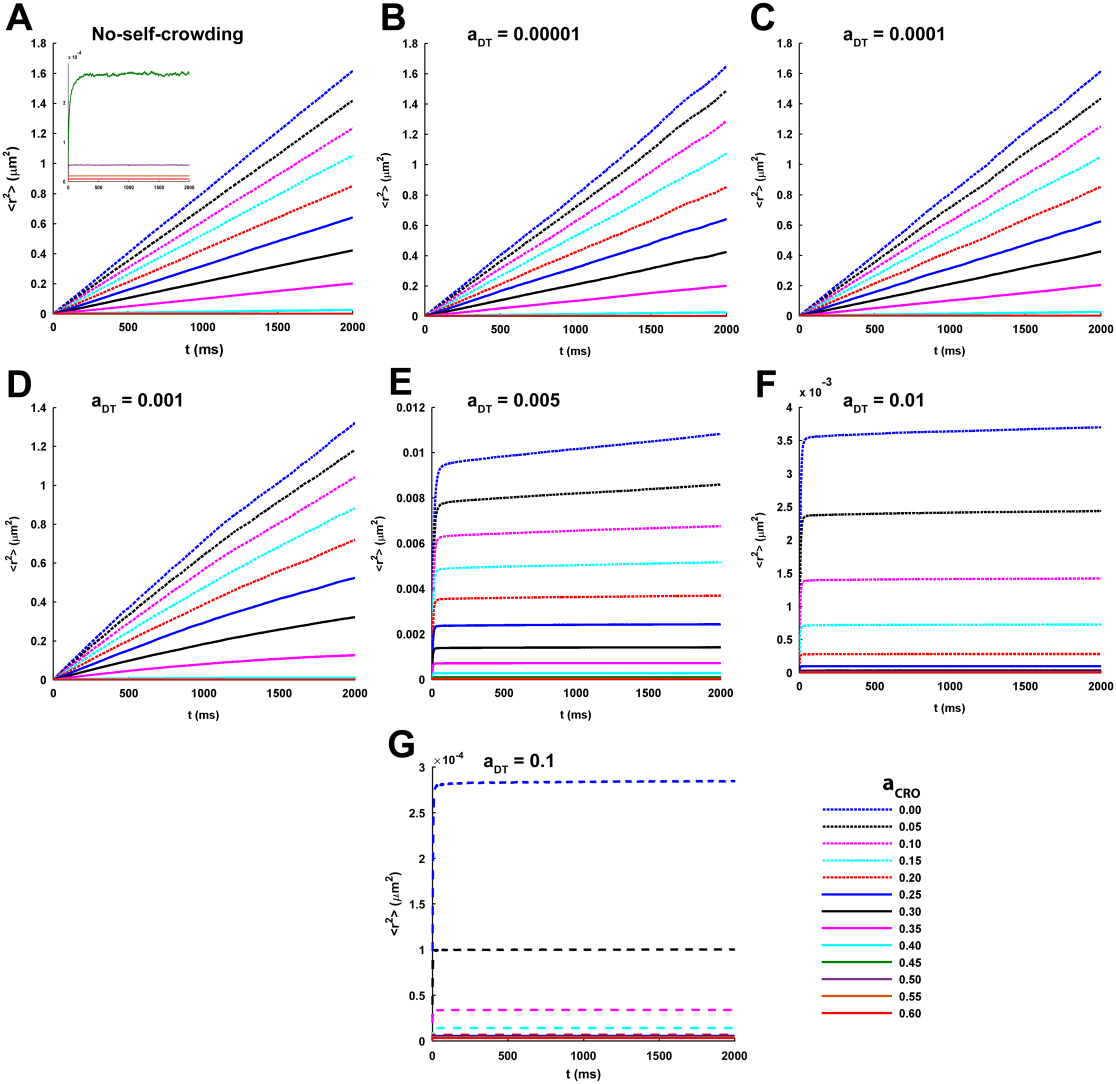
Temporal profiles of ensemble-averaged MSD under different self-crowding conditions and CRO densities. (A-G) Each plot demonstrates the temporal profiles of ensemble-averaged MSD, 〈*r*^2^〉, across the increasing CRO density, *a*_*CRO*_, for a given condition of self-crowding. The no-self-crowding condition refers to the conventional approach where self-crowding of diffusing tracers is not considered in the simulation and, hence, is regarded here as the standard benchmark for comparison. Otherwise, the different self-crowding conditions are recognized by the density of DTs, *a*_*DT*_, on the lattice with which the diffusion simulation is performed. The tracer diffusion appears normal for lower densities of CROs and DTs where 〈*r*^2^〉 appears to increase linearly with time. However, it becomes strongly subdiffusive-anomalous and confined for the higher CRO and DT densities where 〈*r*^2^〉 reaches a saturation or plateau and no significant increase in MSD occurs further with temporal progression.

In the absence of self-crowding factor, the 〈*r*^2^〉 increases linearly with time for the lower *a*_*CRO*_ = 0.00 − 0.25 (Fig 2A). On the other hand, the conditions of very high *a*_*CRO*_ = 0.45 − 0.60 can be clearly recognized by the confined tracer diffusion as the associated 〈*r*^2^〉 rapidly reaches a plateau (Fig 2A, inset) where no further variation in it occurs with progression of time. This range of *a*_*CRO*_ is close to or above the percolation threshold, *θ*_*P*_, of a square lattice framework for tracer diffusion, which is known to be approximately 0.5 for a sufficiently large square lattice [54]. *θ*_*P*_ of a diffusion lattice signifies the area fraction of the lattice occupied by immobile completely-reflecting obstacles at and beyond which the possibility of an infinite percolation cluster to exist vanishes. In other words, there is no way left for a diffusing tracer to diffuse/percolate to extremely large distances over the lattice as the time progresses and, rather, gets trapped in small domains or confinements. Therefore, the trapping of tracers observed here at this range of *a*_*CRO*_ is technically consistent with the concept of *θ*_*P*_ of a square lattice. However, for the intermediate range of *a*_*CRO*_ = 0.30 − 0.40, the 〈*r*^2^〉 initially increases in a nonlinear manner but later adopts a linear profile (Fig 2A).

**Fig 3:**
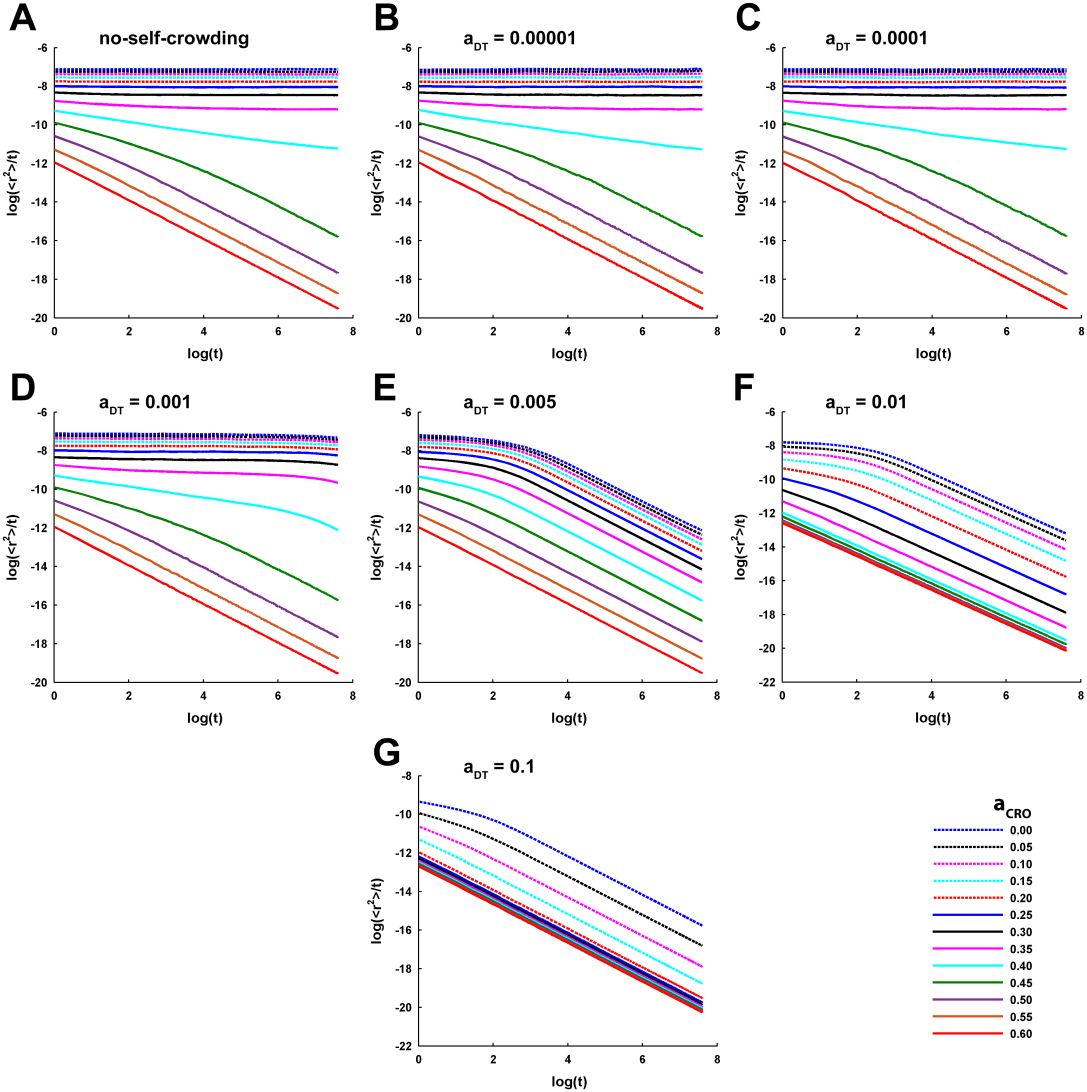
Log-log profiles of the ensemble-averaged MSD under different self-crowding conditions and CRO densities. The profile of variation in natural logarithm of the ratio of ensemble-averaged MSD to time,log (〈*r*^2^〉/*t*), with respect to the natural logarithm of time, log (*t*), is referred here as the log-log profile for simplicity. (A-G) Each plot demonstrates the log-log profiles of MSD across the increasing CRO density for a given condition of self-crowding. Perfectly normal diffusion is characterized by a flat or horizontal log-log profile across the entire duration of MSD measurement. However, profiles exhibiting sharp linear decrease at longer time characterize strongly anomalous sub-diffusion or confined diffusion and indicate the original dependence of MSD on time raised to a fractional power (power-law relation). There are profiles too which show transition from anomalous to normal diffusion. Evidently, tracer diffusion is observed to be confined and strongly anomalous at higher densities of CROs and DTs.

The effect of varying *a*_*CRO*_ on the 〈*r*^2^〉 of tracer diffusion becomes more conspicuous by looking at the log-log profiles (Fig 3A). The log-log plots depict almost flat horizontal profile for the lower *a*_*CRO*_ and indicates perfectly normal diffusion according to Eq 5. However, increase in *a*_*CRO*_ is marked by a brief initial anomalous diffusion of tracers where the log-log profile bears a negative slope (see Eq 5) and a gradual transition to the later normal diffusion. Remarkably, the crossover length, i.e. the 〈*r*^2^〉 traversed by the tracer after which the anomalous diffusion turns into normal diffusion, and the associated crossover time of the transition are observed to increase with rise in *a*_*CRO*_ (Fig 3A). Only at *a*_*CRO*_ closer to or higher than *θ*_*P*_, a long-term anomalous diffusion appears where the log-log profile steeply decreases in a linear fashion at longer time. This, in turn, depicts a power-law time-dependence of 〈*r*^2^〉 (see Eq 4). Here, the crossover length and time approach infinity.

**Fig 4:**
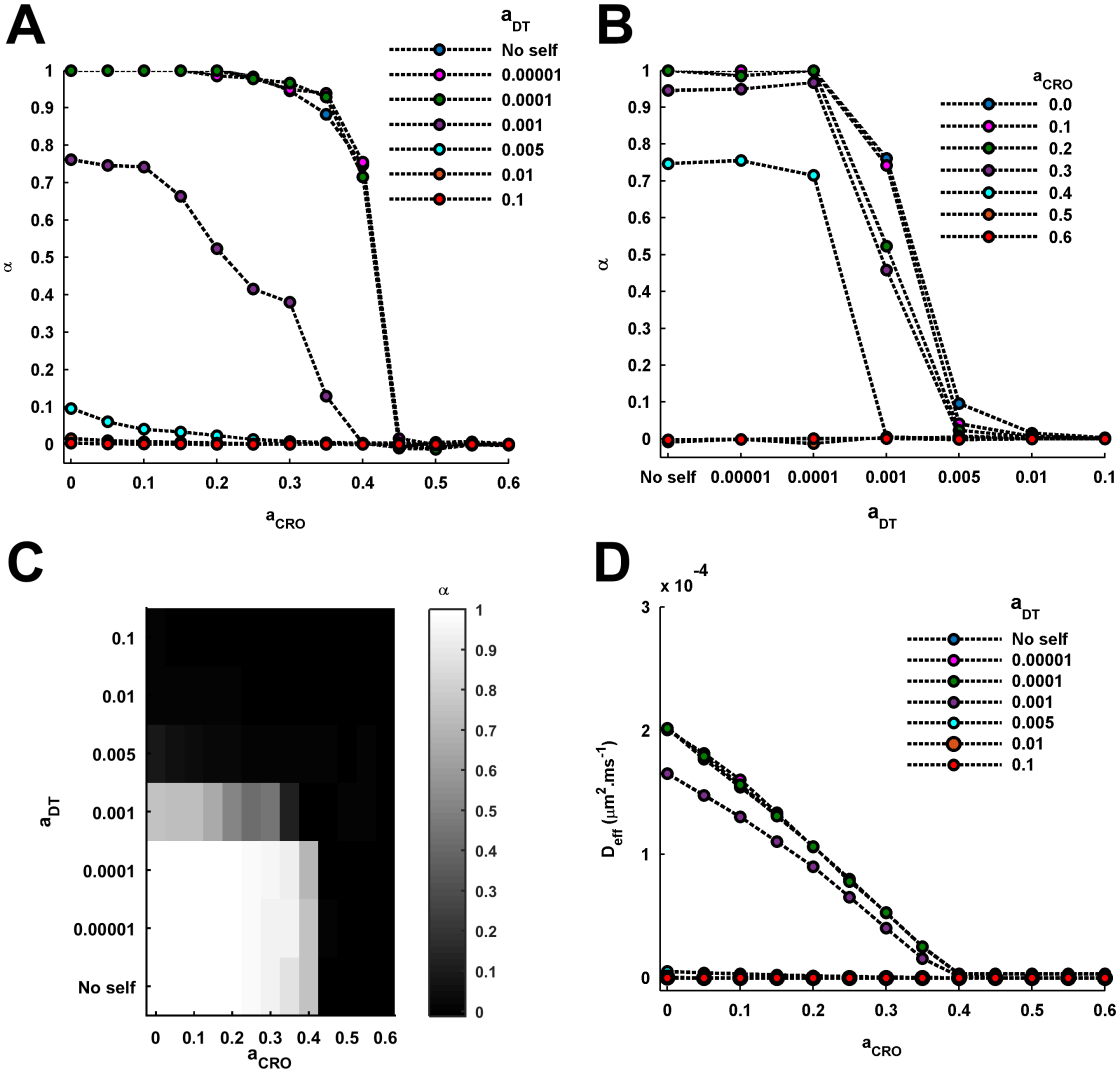
Effect of densities of CROs and DTs on the anomalousity and effective diffusion coefficient of tracer diffusion. Anomalousity of tracer diffusion is characterized through the anomalous exponent, *α*, of the diffusion, which is computed from the log-log profiles of the temporal evolution of MSD. *α* = 1 is associated with perfectly normal diffusion. Lower is the *α*, higher is the anomalousity of tracer diffusion. (A) The variation in *α* across increasing CRO density under different self-crowding conditions is shown. It is apparent that for lower DT densities, the profile of *α* exhibit a switch-like behaviour where the anomalousity steeply rises beyond a certain high level of *a*_*CRO*_ (i.e. 0.4 here) and leads to the strong sub-diffusion or confinement of the diffusing tracers. However, very high levels of *a*_*DT*_ consistently exhibit strong anomalous sub-diffusion regardless of the *a*_*CRO*_. (B) The variation in *α* across increasing *a*_*DT*_ under different conditions of *a*_*CRO*_ is shown. For lower *a*_*CRO*_, the profiles demonstrate a switch-like behaviour across increasing *a*_*DT*_ and there occurs a sudden transition to strongly anomalous diffusion beyond *a*_*DT*_ = 0.001. Increase in *a*_*CRO*_ appears to accentuate the sharpness of the transition. However, for very high *a*_*CRO*_, the tracer diffusion is strongly anomalous regardless of the *a*_*DT*_. (C) The effects of varying *a*_*CRO*_ and *a*_*DT*_ on the anomalousity of tracer diffusion are collectively summarized in the gray-scaled heat-map and is derived from the previous observations made in (A) and (B). It clearly shows that both high CRO density and/or high DT density may lead to strongly anomalous sub-diffusion and trapping of tracers. (D) The variation in effective diffusion coefficient, *D*_*eff*_, of tracer diffusion across increasing CRO density under different self-crowding conditions is shown. For the lower values of *a*_*DT*_ and the no-self-crowding condition, increase in *a*_*CRO*_ leads to a consistent decrease in the *D*_*eff*_. Moreover, their profiles of *D*_*eff*_ are highly overlapping and fairly identical. With increase in *a*_*DT*_, the profile shifts to lower levels, such that very high *a*_*DT*_ is associated with almost negligible *D*_*eff*_ and, in concordance with *α*, indicates severely hampered and confined tracer mobility, regardless of *a*_*CRO*_. Notably, at *a*_*CRO*_ = 0, *D*_*eff*_ of tracer diffusion for no-self-crowding conditions and *a*_*DT*_ ≤ 0.0001 is equivalent to the effective free diffusion coefficient of AMPARs (0.2*nm*^2^.*μs*^−1^) in the extrasynaptic membrane of excitatory synapses.

Using the log-log profiles, the anomalousity of tracer diffusion for the different values of *a*_*CRO*_ is computed in terms of the anomalous exponent, *α*, of 〈*r*^2^〉 using Eq 5. It can be easily noted that the flat horizontal log-log plots for lower *a*_*CRO*_ have slope zero and, thus, *α* = 1. For very high *a*_*CRO*_ characterized with long-term anomalous diffusion of tracers, the slope of the long-time tail of the log-log plots can be easily used to compute *α*, which turns out to be close to or equal to 0. However, for the intermediate values of *a*_*CRO*_ observed with a transition from anomalous to normal diffusion, *α* is computed from the linear fitting to the initial segment of the log-log plot within crossover length associated with anomalous diffusion. Consequently, across the increasing *a*_*CRO*_, the anomalousity of tracer diffusion almost exhibits a sharp inverted sigmoidal profile (Fig 4A). There occurs a sudden decline in *α* (increase in anomalousity) close to *a*_*CRO*_ = 0.4, which has been suggested earlier [31] as the switch-like behaviour leading to the trapping of AMPARs.

Further, using the log-log profiles, *D*_*eff*_ of tracer diffusion is computed under different conditions of *a*_*CRO*_ using Eq 5. The *D*_*eff*_ shows a consistent decrease, unlike *α*, with increase in *a*_*CRO*_ (Fig 4D). This indicates that although diffusion remains normal for lower *a*_*CRO*_, the receptor diffusivity indeed decreases with rise in the crowding conditions of completely reflecting obstacles. Under the extreme conditions of confined diffusion, the *D*_*eff*_ becomes negligible. Altogether, it must be noted that these observations under the standard condition are identical to that reported earlier in a computational study by Santamaria et al. [31].

**Fig 5:**
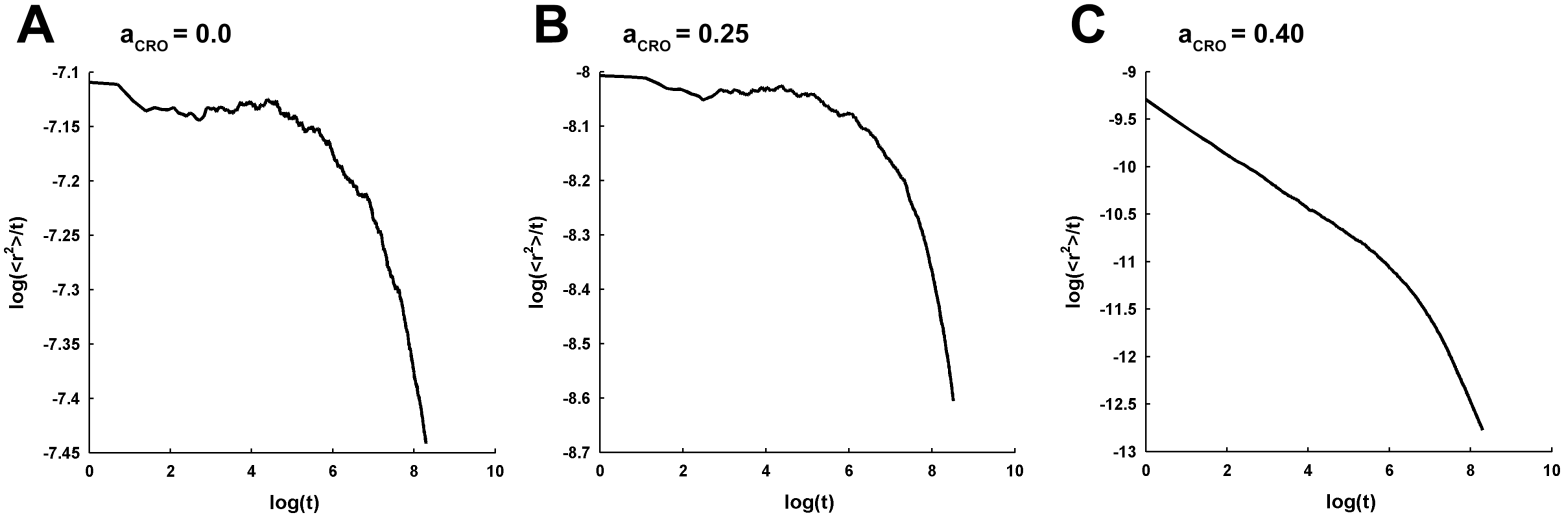
Log-log profiles of the MSD under the self-crowding condition with a_DT_ = 0.001 for an extended duration of 4000ms. The log-log profiles are shown for the select few *a*_*CRO*_ = 0.0 (A), 0.25 (B) and 0.40 (C). It is evident that the profiles exhibit sharp decay at longer time, which was not clearly noticeable in the log-log profiles for the time duration of 2000*ms* shown in Fig 3D.

The temporal profiles of 〈*r*^2^〉 and the associated log-log plots for increasing *a*_*CRO*_ under the additional consideration of the different self-crowding conditions of tracers are shown in Figs 2B–2G and 3B–3G, respectively. Particularly looking at the log-log profiles, it is clear that the self-crowding conditions with *a*_*DT*_ = 0.00001 and 0.0001 (Fig 3B & 3C) exhibit almost an identical behaviour as well as identical to that noted in the no-self-crowding condition (Fig 3A). Even the anomalousity profile across increasing *a*_*CRO*_ under these self-crowding conditions almost mimic that of the no-self-crowding condition (Fig 4A). Therefore, it appears that these self-crowding conditions are associated with sufficiently low tracer density such that they could not noticeably affect the features of tracer diffusion observed under no-self-crowding condition. However, *a*_*DT*_ = 0.001 demonstrates a significant intensity of long-term anomalous diffusion across the entire range of *a*_*CRO*_ (Fig 3D). Although this behaviour does not become clearly visible across the 2*s* measurement time duration, longer time duration of 4*s* makes it clearly visible (Fig 5), where the log-log plots for the selected values of *a*_*CRO*_ exhibit a long-time sharp decay profile. Accordingly, the profile of *α* across increasing *a*_*CRO*_ is significantly affected and shifted to lower levels in comparison to that for the lower *a*_*DT*_ and no-self-crowding condition (Fig 4A). Further, extremely self-crowded conditions with *a*_*DT*_ ≥ 0.005 lead to a very strong long-range anomalous diffusion across all values of *a*_*CRO*_ (Fig 3E–3G) and the entire profile of *α* remains fairly close to zero (Fig 4A).

It can be seen that the range of *a*_*CRO*_ = 0.0 − 0.4 associated with normal receptor diffusion (*α* = 1) is almost invariant (Fig 4A) for the lower *a*_*DT*_ = 0.00001 and 0.0001 and the no-self-crowding condition. Moreover, for these *a*_*DT*_ and the no-self-crowding condition, the window of *a*_*CRO*_ associated with the transition of tracer diffusion from normal to strongly anomalous nature is very narrow. This signifies a sudden rise in anomalousity and a switch-like behaviour for tracer trapping due to high reflecting obstacles’ density. However, for the higher *a*_*DT*_ = 0.001, the range of *a*_*CRO*_ over which perfectly normal diffusion may occur completely vanishes (Fig 4A) and the window of transition from partially normal to strong anomalous diffusion is also very gradual. For *a*_*DT*_ ≥ 0.005, the diffusion remains strongly anomalous irrespective of *a*_*CRO*_ (Fig 4A).

To understand more about how the increase in self-crowding affects the tracer diffusion in the presence of CROs, the *α* is now plotted across the increasing values of *a*_*DT*_ for a given value of *a*_*CRO*_ (Fig 4B). It is interesting to note that intense self-crowding itself may bring strongly anomalous diffusion even in the absence of reflecting obstacles, as observed here for *a*_*DT*_ ≥ 0.005. This contrasts a common fundamental assumption in the earlier theoretical studies [31, 64] that the AMPAR diffusion should be normal in the synaptic membrane in the absence of any non-binding completely-reflecting obstacles. Rather, the results suggest that it may also depend on the AMPAR density in the obstacle-free medium. At the same time, the present observations also support the above assumption to remain valid given the fact that the density of AMPARs in the extrasynaptic membrane is considerably low [4]. Another important thing to be noted is that the profile of *α* across increasing *a*_*DT*_ exhibits a switch like behaviour when *a*_*CRO*_ = 0, akin to that observed above in the case of variation in *a*_*CRO*_ for lower *a*_*DT*_ and no-self-crowding condition (Fig 4B). This switch-like behaviour appears to intensify with increase in *a*_*CRO*_ as the transition becomes sharper. However, for *a*_*CRO*_ ≥ 0.45, the profile is fairly close to or is identically zero across all values of *a*_*DT*_ and the switch-like behaviour completely disappears.

Therefore, in regard of the anomalousity-driven trapping of AMPARs within PSD, self-crowding of AMPARs possibly appears as a new dimension to the causality, which was earlier thought to be driven only by the local macromolecular crowding other than the AMPARs. The cumulative effect of various densities of reflecting obstacles and diffusing tracers on the anomalousity of tracer diffusion observed here is summarized in the heat map shown in Fig 4C. It can be easily noted that, both high *a*_*CRO*_ and/or high *a*_*DT*_ can lead to strongly anomalous confined diffusion of the tracers. Further, the effect of self-crowding on tracer diffusion is distinguishable only at lower or moderate concentrations of reflecting obstacles and increase in *a*_*CRO*_ catalyzes the anomalousity caused by higher *a*_*DT*_. However, for very high CRO concentration, tracer diffusion remains strongly anomalous for all self-crowding and no-self-crowding conditions, owing to the lack of percolation clusters on the 2D square lattice.

To observe the effect of self-crowding on tracer diffusion in terms of the effective diffusion coefficient, *D*_*eff*_ is computed from the log-log profiles under the varying conditions of *a*_*DT*_ and *a*_*CRO*_. As a matter of fact, for diffusion marked with *α* equal to or sufficiently close to 1, the computation of *D*_*eff*_ is very straightforward (see Eq 5). On the other hand, for receptor diffusion marked with *α* equal to or sufficiently close to zero, the *D*_*eff*_ will be certainly negligible as there occurs no apparent diffusion at a substantial timescale. However, for intermediate values of *α*, the diffusion is neither perfectly normal nor completely confined and it becomes difficult to conceive a term like a constant diffusion coefficient to describe the 〈*r*^2^〉 over the entire duration of time. Under such conditions, the diffusion coefficient becomes time-dependent and is generally described through the two kinds of time-dependent quantities viz. apparent diffusion coefficient and the instantaneous diffusion coefficient [67]. The apparent diffusion coefficient, *D*_*app*_, is a time-averaged quantity and signifies the *D*_*eff*_ of normal diffusion which could efficiently lead to the identical 〈*r*^2^〉 at a given time which one gets through the anomalous diffusion. This is given as,

$$\begin{array}{c} D_{app}(t)=\frac{D}{t^{1-\alpha }} \end{array}$$ (6)

Here, *D* is the original constant present in the Eq 4. On the other hand, the instantaneous diffusion coefficient, *D*_*inst*_, represents the instantaneous rate of change of slope of the nonlinear increase in 〈*r*^2^〉 at a given time, which is given as,

$$\begin{array}{c} D_{inst}(t)=\frac{\alpha D}{t^{1-\alpha }} \end{array}$$ (7)

As evident, both the quantities decrease with progression of time in anomalous diffusion [67].

For the case here, use of *D*_*app*_ is more suitable as it provides a sense of effective diffusion coefficient which could be used to describe diffusion conditions characterized with the intermediate values of *α* between zero and one. However, the choice of *D*_*app*_ would necessarily depend on the time duration for which the process is observed. In the earlier studies [24, 29, 31], distribution of diffusion coefficient is also shown and the statistical parameters such as median diffusion coefficient is computed. Yet, there also the distribution is strictly dependent on the time at which the observation is made and the statistical parameters do temporally evolve. Therefore, the *D*_*app*_ is computed for the time point of 2*s*, which is the time duration of diffusion measurement performed in the present study, and will be considered here as the *D*_*eff*_ of tracer diffusion characterized with intermediate values of *α*.

For *a*_*DT*_ = 0.00001 and 0.0001, the variation in *D*_*eff*_ with increase in *a*_*CRO*_ is completely overlapping with that for the no-self-crowding condition (Fig 4D) and, accordingly, the tracers mobility gradually decreases with increase in *a*_*CRO*_. However, the profile for *a*_*DT*_ = 0.001 is shifted to slightly lower values depicting reduced mobility due to increased self-crowding of the receptors. Indeed, in this case too, the mobility appears to decrease with increase in *a*_*CRO*_. For the rest very high values of *a*_*DT*_ ≥ 0.005, the entire profile of *D*_*eff*_ is shifted to extraordinarily low levels (Fig 4D) depicting heavily hampered mobility of tracers owing to steric-exclusion and confinement among themselves as well as in the presence of CROs. Altogether, high density of completely reflecting obstacles and/or tracers engenders reduced mobility and confinement in terms of both the anomalousity as well as effective diffusion coefficient of the tracer diffusion.

In this regard, the earlier experimental studies involving monitoring of the properties of a diffusing entity in the presence of same entity acting as the crowders also appears to strongly corroborate the above observations resulting from the self-crowding. A recent study by Roosen-Runge et al. [68] on the diffusion of bovine serum albumin in the aqueous solution using neutron backscattering has revealed that increase in the volume fraction occupied by the protein (even upto 30%) causes strong decline in the translational diffusion coefficient and leads to shorter-time self-diffusion, implying anomalous nature in action. Similarly, another experimental study by Ramadurai et al. [69] involving fluorescence correlation spectroscopy of the lateral diffusion of a variety of integral transmembrane proteins of different sizes, such as monomeric LacY to trimeric glutamate transporters, at their different density on artificially reconstituted large lipid vesicles demonstrates that increase in the size and density of the subject protein leads to strong decline in the later diffusion coefficient. Further, it has been shown that there occurs a significant decrease in the anomalous exponent of the diffusion at sufficiently high density of the proteins and is evitable even for monomeric proteins, such as LacS. A very recent study by Houser et al. [70] on the lateral diffusion of a homogeneous population of transferrin membrane proteins using fluorescence correlation spectroscopy has also shown that increase in the membrane coverage by the protein leads to strong decline in the diffusivity and has emphasized on the steric-exclusion underlying the self-crowding of the protein. It must be noted that transferrin occupy much lesser membrane area (∼ 24*nm*^2^) in comparison of our subject protein, AMPAR.

Therefore, the self-crowding of bulky AMPARs implied here through the tracer diffusion indeed appears to be a significant factor at play in the anomalous diffusion and trapping of these receptors in the PSD, where these receptors are generally present at high density. The SI S1 Video demonstrates the temporal evolution of the position of a diffusing tracer under different self-crowding conditions, but in the absence of any other obstacles, as well as a control condition of free-diffusion.

### Features of tracer diffusion in the presence of binding submembranous obstacles

Three levels of uniform binding energies representing weak (2*k*_*B*_*T*), intermediate (6*k*_*B*_*T*) and strong (10*k*_*B*_*T*) binding of tracers to the binding obstacles (PROs) are separately considered. Given a binding energy, four arbitrary densities of PROs, *a*_*PRO*_ = 0.2, 0.4, 0.6 and 0.8, over the lattice are sampled to broadly capture the different situations of the accumulation of scaffold proteins, ranging from sparse to very dense, underneath the PSD. Subsequently, these combinations are examined for the different conditions of self-crowding of tracers. In this part of the study, reflecting obstacles are completely absent and only the role of binding obstacles in shaping the nature of tracer diffusion is examined.

**Fig 6:**
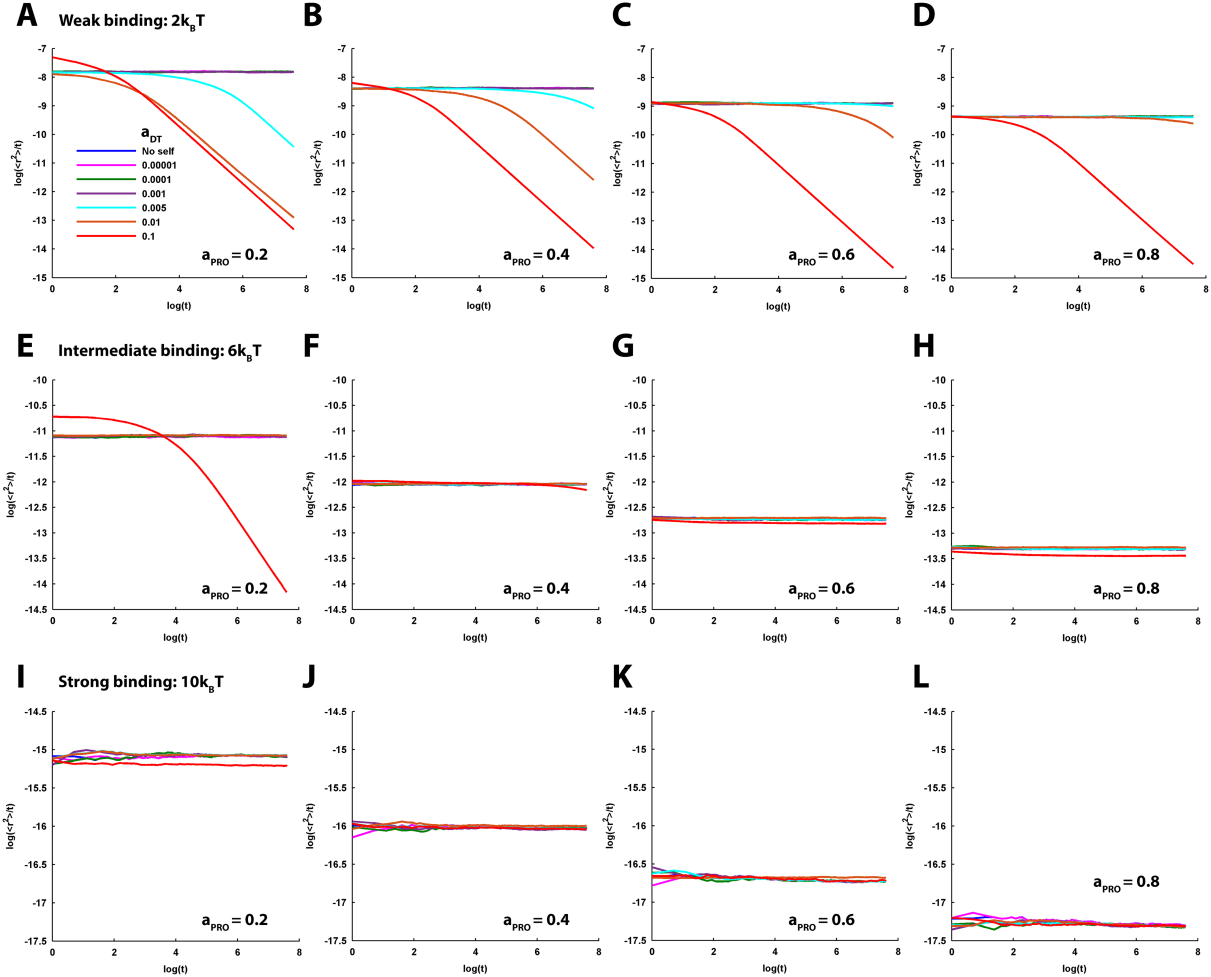
Log-log profiles of the MSD of tracer diffusion for the varying DT-PRO binding energy and PRO density under different self-crowding conditions. Three levels of DT-PRO binding energy, 2, 6 and 10 *k*_*B*_*T*, are considered to represent the situations of weak, intermediate and strong DT-PRO binding, respectively. Four increasing PRO densities viz. *a*_*PRO*_ = 0.2, 0.4, 0.6 and 0.8 are sampled to account for a range of sparse to dense submembranous crowding of PROs at the PSD. Subsequently, these combinations are examined for the different conditions of self-crowding. Here, the log-log profiles under the different conditions of self-crowding are laid together in a single plot. (A-D) Weak -PRO binding. The profiles for no-self-crowding condition and *a*_*DT*_ ≤ 0.001 are consistently flat (representing perfectly normal diffusion) regardless of the *a*_*PRO*_ as well as fairly overlap with each other. For *a*_*DT*_ ≥ 0.005, the profiles indicate strong anomalous diffusion for the lower *a*_*PRO*_ = 0.2. Increase in *a*_*PRO*_ causes a gradual transition of the profiles from strongly anomalous to normal diffusion, though the intensity of the anomalousity relaxation further depends on the level of *a*_*DT*_. For example, the profile for *a*_*DT*_ = 0.005 acquires perfectly normal behaviour by *a*_*PRO*_ = 0.8 and overlaps with that of the lower *a*_*DT*_. However, the profile for *a*_*DT*_ = 0.01 carries slightly anomalous behaviour even at *a*_*PRO*_ = 0.8. The anomalousity in the profile for *a*_*DT*_ = 0.01 remains insignificantly affected by the increase in *a*_*PRO*_. (E-H) Intermediate DT-PRO binding. The profiles for the no-self-crowding condition and *a*_*DT*_ ≤ 0.01 consistently exhibit normal diffusion regardless of *a*_*PRO*_ and fairly overlap with each other. Notably, *a*_*DT*_ = 0.1 is associated with strongly anomalous behavior for the lower *a*_*PRO*_ = 0.2. However, the anomalousity steeply decreases with the increase in *a*_*PRO*_ and the log-log profile approaches closer to that for the *a*_*DT*_ ≤ 0.01. (I-L) Strong DT-PRO binding. The profiles for the no-self-crowding condition and all values of *a*_*DT*_ consistently exhibit normal diffusion regardless of the *a*_*PRO*_ and remain sufficiently overlapping with each other. (A-L) Altogether, increase in binding ameliorates anomalousity of tracer diffusion arising from their higher self-crowding. Furthermore, increase in *a*_*PRO*_ and binding energy cause the log-log profiles to shift to lower levels. In general, this has implications to the decrease in tracer mobility in terms of the effective diffusion coefficient.

**Fig 7:**
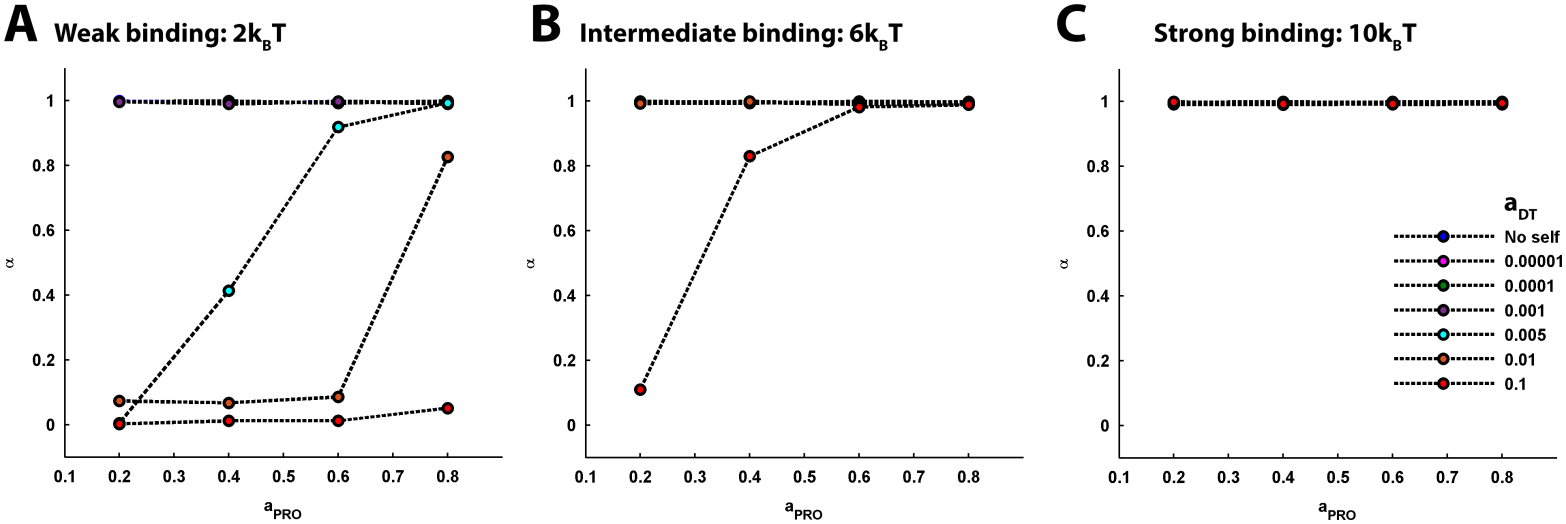
The effect of varying DT-PRO binding energy and PRO density on the anomalousity of tracer diffusion, under different self-crowding conditions. (A) Weak DT-PRO binding. The profiles of variation in anomalous exponent, *α*, of the tracer diffusion under the different conditions of self-crowding are plotted together across the increasing *a*_*PRO*_. *α* remains equal to one across the entire span of *a*_*PRO*_ for the no-self-crowding condition and the self-crowding conditions with *a*_*DT*_ ≤ 0.001. For *a*_*DT*_ = 0.005, *α* gradually rises from zero and approaches 1 with increase in *a*_*PRO*_. However, the rise in *α* becomes significantly slower for the higher *a*_*DT*_ = 0.01, such that it remains less than 1 even at *a*_*PRO*_ = 0.8. In continuation, for very high *a*_*DT*_ = 0.1, effect of increasing *a*_*PRO*_ is visibly insignificant and *α* remains close to zero. (B) Intermediate DT-PRO binding. *α* remains 1 across the entire span of *a*_*PRO*_ for the no-self-crowding condition and the self-crowding conditions with *a*_*DT*_ ≤ 0.01. It is only for *a*_*DT*_ = 0.1 that, with increase in *a*_*PRO*_, *α* sharply rises and approaches 1 by *a*_*PRO*_ = 0.6. (C) Strong DT-PRO binding. *α* remains 1 across the entire span of *a*_*PRO*_ for the no-self-crowding condition as well as all values of *a*_*DT*_. (A-C) It is evident that increase in binding reduces anomalousity of tracer diffusion arising from self-crowding. However, the intensity of *a*_*PRO*_-dependent amelioration of anomalousity further depends on the intensity of self-crowding.

**Fig 8:**
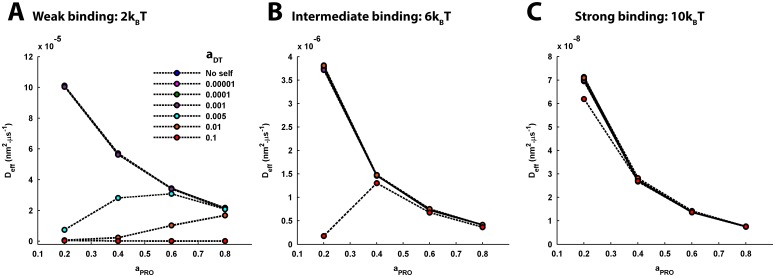
The effect of varying DT-PRO binding energy and PRO density on the effective diffusion coefficient of tracer diffusion, under different self-crowding conditions. (A) Weak DT-PRO binding. For the no-self-crowding condition and self-crowding conditions with *a*_*DT*_ ≤ 0.001, the effective diffusion coefficient, *D*_*eff*_, of tracer diffusion appears to consistently decrease with increase in *a*_*PRO*_ and their profiles also fairly overlap with each other. For *a*_*DT*_ = 0.005, *D*_*eff*_ initially rises with increase in *a*_*PRO*_ and closely approaches the *D*_*eff*_ for lower *a*_*DT*_ at *a*_*PRO*_ = 0.6. Subsequently, following the *D*_*eff*_ profile for lower *a*_*DT*_, it decreases with further increase in *a*_*PRO*_. For *a*_*DT*_ = 0.01, *D*_*eff*_ gradually rises with increase in *a*_*PRO*_ and closely approaches the profile of variation in *D*_*eff*_ for lower *a*_*DT*_ by *a*_*PRO*_ = 0.8. The rise in *D*_*eff*_ observed here with rising *a*_*PRO*_ is owing to the associated reduction in anomalousity and confinement of tracer diffusion. However, for *a*_*DT*_ = 0.1, *D*_*eff*_ remains significantly low across the entire span of *a*_*PRO*_. (B) Intermediate DT-PRO binding. *D*_*eff*_ appears to consistently decrease with increase in *a*_*PRO*_ for the no-self-crowding condition and self-crowding conditions with *a*_*DT*_ ≤ 0.01. However, for *a*_*DT*_ = 0.1, *D*_*eff*_ sharply rises with increase in *a*_*PRO*_ and closely approaches the profile of variation in *D*_*eff*_ for lower *a*_*DT*_ by *a*_*PRO*_ = 0.4. Further increase in *a*_*PRO*_ leads to gradual decrease in *D*_*eff*_. (C) Strong DT-PRO binding. *D*_*eff*_ consistently decreases with increase in *a*_*PRO*_ for the no-self-crowding condition as well as all values of *a*_*DT*_. Further, the profiles of the variations in *D*_*eff*_ are sufficiently overlapping. (A-C) It is evident that the scale of the magnitude of *D*_*eff*_ significantly decreases with increase in the DT-PRO binding energy. Accordingly, increase in binding, either through increase in binding energy or PRO density or both, in general leads to reduction in tracer mobility. Only for the conditions of self-crowding where increase in binding leads to reduction in anomalousity of tracer diffusion, one may observe a relative increase in *D*_*eff*_.

The features of tracer diffusion under no-self-crowding condition is surely monotonous in the presence of binding obstacles. The tracer diffusion is always perfectly normal for all binding energies and values of *a*_*PRO*_, as the log-log profiles (Fig 6) remains fairly horizontal along the entire duration of diffusion monitoring and the *α* remains strictly close to one (Fig 7). However, for a given binding energy, the log-log profile shifts to lower values with increase in *a*_*PRO*_. Increase in binding energy further lowers the levels of these log-log profiles. This has implications in the decline of tracer mobility in terms of *D*_*eff*_. Accordingly, the *D*_*eff*_ exponentially decreases with increase in *a*_*PRO*_ for a given binding intensity (Fig 8). Moreover, increase in binding intensity shifts the *D*_*eff*_ profile to lower orders of magnitude, depicting further decline in tracer mobility. These observations for no-self-crowding condition are equivalent to that observed in the computational study by Sanatamaria et al. [31]. Further, the absence of anomalousity in tracer diffusion in the presence of a wide range of PRO density and binding energy is also consistent with the previous study of anomalous diffusion in the presence of binding performed by Saxton [55], where it is implied that simple valley models of tracer binding always leads to normal diffusion under thermally-equilibrated initial condition.

The self-crowding conditions with *a*_*DT*_ = 0.00001, 0.0001 and 0.001, are found to exhibit behaviors identical to that under the no-self-crowding condition. For a given binding energy and *a*_*PRO*_, the log-log plots across these self-crowding conditions are strongly overlapping with that of the no-self-crowding condition (Fig 6). Accordingly, diffusion is normal across all the values of *a*_*PRO*_ and the levels of binding energies with *α* close to one (Fig 7). Further, the *D*_*eff*_ for these self-crowding conditions demonstrate a consistent decrease in the tracer mobility with increase in binding energy and PRO density (Fig 7). Also, the *D*_*eff*_ profiles are sufficiently overlapping for these conditions of self-crowding as well as no-self-crowding. Therefore, it appears that the increase in tracer density to 0.001 has no distinguishable effect on the tracer diffusion in the presence of binding obstacles. Rather the diffusion is being mainly governed by the PRO density and the binding energy.

On the other hand, the self-crowding conditions with *a*_*DT*_ ≥ 0.005 exhibit a peculiar behaviour. For weak binding events, these self-crowding conditions clearly demonstrate a strong long-range anomalous diffusion for lower PRO density, *a*_*PRO*_ = 0.2, (Fig 6A) and the values of *α* are close to zero (Fig 7A). However, as the PRO density is increased, the anomalousity of diffusion gradually reduces and the log-log profiles tend to approach normal diffusion behaviour. For the case of *a*_*DT*_ = 0.005, diffusion becomes fairly normal at *a*_*PRO*_ = 0.8 (Fig 6D) and *α* reaches 1 (Fig 7A). Tracer diffusion for *a*_*DT*_ = 0.01 also tends to acquire normal behaviour with rising *a*_*PRO*_, though there remains slight anomalousity even at *a*_*PRO*_ = 0.8. However, for = 0.1, the diffusion remains strongly anomalous even at = 0.8 (Figs 6D & 7A). An important thing to observe is that the log-log profile of normal diffusion that the anomalous tracer diffusion for *a*_*DT*_ = 0.005 and 0.01 gradually approaches (Fig 6D)with increase in *a*_*PRO*_ appears to overlap with that obtained for *a*_*DT*_ ≤ 0.001 as well as for the no-self-crowding condition.

Remarkably, *a*_*DT*_ = 0.005 and 0.01 consistently exhibit normal diffusion across all PRO densities for the intermediate and strong binding energies, as their log-log plots (Fig 6E–6L) remain horizontal with *α* = 1 (Fig 7B & 7C). Moreover, these log-log plots fairly overlap with that of the lower self-crowding conditions under the respective conditions of binding energies and *a*_*PRO*_. However, for the intermediate binding intensity, *a*_*DT*_ = 0.1 exhibits significant anomalous diffusion for lower *a*_*PRO*_ = 0.2 (Figs 6E & 7B). The anomalousity soon vanishes for *a*_*PRO*_ ≥ 0.6 (Fig 6G & 6H) and *α* reaches 1 (Fig 7B). For strong binding intensity, *a*_*DT*_ = 0.1 exhibits perfectly normal diffusion for all values of *a*_*PRO*_ and identical to the lower self-crowding conditions (Figs 6I–6L & 7C).

At this point, if we remind the observations regarding tracer diffusion in obstacle-free medium, *a*_*DT*_ ≥ 0.001 demonstrated a marked long-range anomalous diffusion (Fig 3D–3G) with significantly low *α* (Fig 5A–5C). Together with the observations made here in the presence of binding obstacles, it is strongly evident that increase in binding phenomenon, either through increase in PRO density or/and increase in binding energy, reduces the anomalousity in tracer diffusion arising from higher self-crowding. However, it is also observed that the intensity of amelioration of the anomalousity with increase in binding further depends on the intensity of self-crowding. Very intense self-crowding conditions would require a considerably large increase in binding energy and scaffold density to exhibit perfectly normal diffusion. As shown here, for strong tracer-PRO binding, receptor diffusion is completely governed by binding obstacles’ density, regardless of the self-crowding conditions.

Nonetheless, for weak binding intensity, tracers mobility in terms of *D*_*eff*_ for *a*_*DT*_ = 0.005 initially increases with increase in *a*_*PRO*_ and later decreases along the profiles obtained for lower *a*_*DT*_ (Fig 8A). However, for *a*_*DT*_ = 0.01, *D*_*eff*_ consistently increases with increase in *a*_*PRO*_. The increase in *D*_*eff*_ under these conditions of *a*_*DT*_ owes to the concomitant relaxation of anomalousity of tracer diffusion. For *a*_*DT*_ = 0.1, *D*_*eff*_ remains significantly close to zero for all values of *a*_*PRO*_ (Fig 8A) due to strongly anomalous tracer diffusion. For intermediate binding intensity, the profile of variation in *D*_*eff*_ for *a*_*DT*_ = 0.005 and 0.01 is identical to that of the lower self-crowding as well as no-self-crowding conditions (Fig 8B). However, for *a*_*DT*_ = 0.1, *D*_*eff*_ sharply rises with increase in *a*_*PRO*_ but soon gets along the decreasing profiles obtained for lower values of *a*_*DT*_. For the strong binding intensity, all conditions of *a*_*DT*_ exhibit an identical profile of decrease in *D*_*eff*_ with rise in *a*_*PRO*_ (Fig 8C).

Therefore, increase in binding indeed ameliorates anomalousity of tracer diffusion arising from the self-crowding of the tracers and stronger binding favors normal diffusion even under high tracer density. However, in regard of *D*_*eff*_, increase in binding consistently reduces the mobility for low tracer density. But for high tracer density, increase in binding leads to higher mobility in the situation where concomitant reduction in the diffusion-associated anomalousity is observed. Otherwise, given a sufficiently strong binding condition, any further increase in binding leads to consistent reduction in the tracers mobility.

## Discussion

The present study deals with the aspect of how self-crowding of mobile bulky AMPARs may affect their lateral diffusion at different densities of the receptors in the postsynaptic membrane of the excitatory synapses and the way in which presence of obstacles and binding elements in the PSD may further influence the effect of self-crowding. In light of the above observations obtained through the Monte-Carlo simulation using the lattice model of diffusion of the representative point tracers, it would be reasonable to state that the density of AMPARs may significantly influence the nature of their diffusion and very high density may lead to strongly anomalous confined diffusion even in the absence of any other obstacles in the membrane. The presence of other transmembrane obstacles may further accentuate the appearance of anomalousity arising from the self-crowding of the receptors. Conversely, self-crowding may cooperate in the trapping of AMPARs effectuated by the intense crowding of transmembrane proteins in the PSD. However, partially-reflecting and binding scaffold proteins lying submembranously within PSD region may serve a contrary role where increase in binding, either through increase in the density of scaffold proteins or increase in the AMPAR-scaffold binding energy or both, reduces the anomalousity in receptor diffusion arising from their self-crowding. Therefore, in the context of anomalousity, the transmembrane obstacles and the binding submembranous scaffold may behave as the two opposing forces.

In the context of effective diffusion coefficient of the AMPARs, the receptor mobility may strongly decrease with rise in the self-crowding of the receptors, in concordance with the increase in anomalousity. And, the presence of transmembrane obstacles may further lead to the decrease in *D*_*eff*_. However, binding may have differential impacts on the receptor mobility and it depends on the intensity of self-crowding. For low and moderate intensity of self-crowding, increase in binding may consistently lead to decrease in the receptor mobility. However, for intense self-crowding conditions involving strong anomalousity of receptor diffusion, increase in binding may increase receptor mobility as long as it is associated with the reduction of anomalousity. Otherwise, once the diffusion acquires a normal behavior under sufficiently strong binding conditions, further increase in binding may lead to decline in the *D*_*eff*_. Yet, despite this differential behavior, binding can be considered to cause, in general, decrease in receptor mobility in terms of its *D*_*eff*_.

Noting these contrary implications of binding in anomalousity and effective mobility of the receptor, a genuine concern arises that which among these two features matters the most in regard of hampering the receptor diffusion. Indeed, it is the anomalousity which marks the most dominant contribution to declined receptor diffusion. If the diffusion is normal, a particle is at least able to diffuse (the MSD grows consistently with time) and escape a defined region of interest, no matter slowly due to a low diffusion coefficient. But in the case of anomalous diffusion, the *D*_*eff*_ continues to decline to zero as the time progresses (see Eq 6) and the amount of decrease in *D*_*eff*_ is directly proportional to the decrease in the anomalous exponent of the diffusion [66, 67]. Accordingly, it is possible that the particle may never escape a defined region under strongly anomalous condition. This is the reason that a diffusing particle exhibiting small anomalous exponent is widely referred to as confined within a region of certain confinement length, as the MSD comes to settle down at a plateau with time. Therefore, it must be recognized that, although binding reduces the receptor mobility, it certainly assists in bringing out the diffusing receptors from the more restricting situation of anomalous diffusion arising from their self-crowding and getting trapped.

### Plausible mechanisms underlying the effects of obstacles on the self-crowded diffusion of diffusing tracers

In the case of obstacle-free diffusion of tracers, increase in the tracer density is responsible for the more frequent self-crowding collisions among the tracers and the resulting obstructions of their diffusion paths. Through the above observations, it is realized that, for the given specifications of the lattice dimension, a rise in tracer density (*a*_*DT*_) to the order of 0.001 commences the appearance of anomalousity in tracer diffusion and further increase in the tracer density leads to its more noticeable magnitude. The appearance of anomalousity can easily be better portrayed under an assumed condition of extreme self-crowding when *a*_*DT*_ is sufficiently close to 1 and almost all lattice sites are occupied with the tracers. At every time-step of simulation, the hopping of a tracer to any random direction would be denied because the neighboring destination sites in almost all directions are occupied with the tracers. This will repeatedly occur across sampling of the entire population of tracers and, as a consequence, the tracers would remain stuck at their positions along a unit advancement in time. This condition would remain unchanged for every further time-steps and the MSD would not increase with time, depicting extraordinarily strict confinement of the tracers. It can now be extrapolated for the lower tracer densities that the confinement would be certainly reduced but the abundance of restricted diffusion would accordingly lead to anomalousity. Need not to say that, for the no-self-crowding condition, diffusion of single tracer on the obstacle-free lattice would always remain perfectly normal.

When reflecting obstacles (CROs) are added to the system, the unoccupied fraction of the lattice sites connected to each other through the diffusive edges decreases. In fact, this decrease in percolation paths is significant only when the CRO density reaches the percolation threshold of the square lattice. This is the reason that, for the conditions of single tracer diffusing in the lattice frame with no self-crowding at all or low tracer density with insignificant counts of self-obstructions during diffusion, what only shapes the nature of tracer diffusion is the extent to which CRO density is close to or beyond the percolation threshold. However, when tracer density is sufficient to effectuate a considerable amount of self-crowding against their free diffusion, slight decrease in percolation paths even at much lower CRO density can exacerbate the anomalousity of diffusion arising from the self-crowding. Furthering this description to the conditions of extreme self-crowding at very high tracer density, a situation appears where even in the absence of reflecting obstacles, the anomalousity of tracer diffusion is close to its possible maximum level and adding reflecting obstacles does not manifest into any significant change.

Unlike the above cases, stating the exact mechanism involved in the observed effects of increase in binding on the self-crowded diffusion of the tracers is not so straightforward. Therefore, the attempt here would be to carefully and systematically deduce the plausible mechanism, while keeping in mind the specific arrangements utilized in the above simulation experiments and the features associated with them in the background. If *a*_*PRO*_ is the fraction of lattice sites occupied by binding obstacles (PROs), (1 − *a*_*PRO*_) is the fraction unoccupied by them. It essentially results into a partition of the lattice medium into two spatial subsets viz. non-binding and binding spatial subsets. The latter is capable of binding a diffusing tracer and freezing it at its location for a random size of waiting time. Notably, the mean waiting time is directly proportional to the intensity of binding such that higher is the binding energy, longer is the mean waiting time. Another important thing to note is the size of binding subset relative to the non-binding subset. Higher is the *a*_*PRO*_, larger is the binding subset.

At the beginning of the simulation, when tracers are uniformly distributed over the lattice with the desired area fraction *a*_*DT*_, *a*_*DT*_ *a*_*PRO*_ would be the fraction of *a*_*DT*_ lying on the PROs and, thus, lying in the binding spatial subset whereas *a*_*DT*_(1 − *a*_*PRO*_) will be lying on completely empty nascent lattice sites and, thus, belongs to the non-binding spatial subset. As the lateral diffusion proceeds, there occurs diffusion of tracers within their own spatial subsets as well as diffusion-associated exchange of tracers across the subsets. Due to reduced tracer mobility in the binding subset, there would occur an initial drift of a certain fraction of the tracer population belonging to the non-binding subset towards the binding subset acting as a sink, until a thermal equilibrium is achieved. Higher is the binding energy and larger is the binding subset, the thermal equilibrium would be acquired with a larger fraction drifted. Once such an equilibrium distribution of the tracer population between the two spatial subsets is achieved, contribution of each population to the anomalousity of entire tracer diffusion can be easily compartmentalized and examined.

The process of equilibrium distribution engenders two consequences for the tracers belonging to the non-binding spatial subset. First, the resultant density of tracers within the non-binding subset is significantly reduced leading to a reduced self-crowding condition. Second, the binding subset-associated larger population of tracers appear as almost static reflecting obstacles (CROs) to the highly mobile tracers belonging to the non-binding subset. Here comes the role of longer mean waiting time under stronger binding condition which leads to larger decline in the hopping rate of the tracers belonging to the binding spatial subset. Therefore, under strong binding conditions, the entire diffusion system for the tracers associated with non-binding spatial subset turns into the diffusion of tracers at low density but in the presence of less or moderately dense CROs. And, according to the previous experiences with the reflecting obstacles, the contribution to anomalousity from the diffusion of unbound tracers is severely reduced. One can now envisage that decrease in binding will certainly violate this setup by bringing more self-crowding encounters amongst the tracers and their resulting anomalousity would be higher.

On the other hand, the diffusion of tracer population belonging to the binding subset within its own spatial subset appears, according to the results, less anomalous under stronger binding conditions. It seems that declined rate of hopping is beneficial in reducing anomalousity by frequently avoiding self-crowding encounters. This is even helpful for the case of encounters with highly mobile tracers belonging to the non-binding spatial subset, which are themselves in lesser density too. However, binding certainly reduces the mobility of the tracers belonging to binding spatial subset. Corollary, lesser and weaker binding would increase the tracers mobility but would concomitantly cause more frequent self-crowding encounters within the binding spatial subset as well as across the non-binding subset and lead to higher anomalousity. This entire description of the possible mechanism concludes at one interesting fact that self-crowding collisions are the main source of anomalousity. Although binding reduces the effective mobility of the tracers, it ameliorates anomalousity by avoiding such collisions. Therefore, the phenomenon of binding plays its role at a trade-off point between the effective mobility of the tracers and the anomalousity of their diffusion.

### Relevance of point tracer diffusion to the diffusion of bulky AMPARs

In the present study, the diffusing tracers are point particles diffusing on the lattice framework. Therefore, one may argue over how the self-crowded diffusion of point tracers may capture the crowded diffusion of AMPARs, which are bulky transmembrane structures with non-zero lateral span. The reply to this question is hidden in the description of area-fraction of the lattice occupied by the point tracers and the use of ensemble-averaged MSD. The extracellular domain of an AMPAR is the most bulky structure with lateral dimensions of length 16*nm* and width 8*nm* [35, 42]. Therefore, its two-dimensional projection on the lipid membrane would occupy a surface area of roughly 128*nm*^2^. For the purpose of realizing side-ways collisions during lateral diffusion, the complex details of an AMPAR structure can be essentially reduced to a transmembrane cylindrical structure [29] of radial width 6*nm*. This lateral radial span characterizes the exclusion area (128*nm*^2^) which avoids approach of another receptor closer than this radius and presumably reflects it away in an elastic manner. Certainly, association of the receptor with other auxiliary proteins [49–51] would further stretch the exclusion area, as it becomes more bulky along the lateral dimension. Given the density of the AMPARs and the areal span of the PSD or a subregion within the PSD, one may easily procure the resultant fraction of the PSD area occupied by the total exclusion area of the receptor population. This fraction amounts to the area-fraction of the self-obstructing point tracers, *a*_*DT*_, on the lattice referred here. Nevertheless, this approach gets complete only when ensemble-averaged MSD of the tracers is used to capture the bulk diffusion properties of AMPARs. Had it been time-averaged MSD observation of single tracers, the statistical approximation using *a*_*DT*_ would not suffice to fully reproduce the time-averaged MSD behaviour of the non-zero size AMPARs [71].

For instance, the density of AMPARs in the extrasynaptic membrane has been experimentally measured to be 3–5*μm*^−2^ [4]. The estimated length scale of the region of extrasynaptic membrane on the spine head is approximately 1*μm* [72] and an effective surface area close to 1*μm*^2^. Therefore, the fraction of extrasynaptic region occupied by the total exclusion area of the receptor population would be 0.00038 − 0.00064. Given this fraction as *a*_*DT*_ in the present study, it is shown that the tracer diffusion would be perfectly normal in the absence of any obstacle. The same is observed in the particle tracking experiments [24–29] and the receptor diffusion is normal in the extrasynaptic membrane, which contains least transmembrane obstacles [27]. However, within the PSD of typical radial size 100*nm* [72, 73], the AMPAR count may range from 20–100 [74] which is equivalent to receptor density 650–3000*μm*^−2^ [74, 75]. These estimates lead to an area fraction of 0.08 − 0.4 of the PSD to be occupied by the total exclusion area of the receptor population. Given this fraction as *a*_*DT*_, it is shown here that self-crowding of the tracers would immensely contribute to the anomalousity of tracer diffusion and their confinements.

Nonetheless, the convergence of lattice model of diffusion under the described conditions of reflecting and binding obstacles to continuous-space diffusion becomes important to be investigated. In regard of the earlier studies on AMPAR diffusion in the absence of self-crowding interactions amongst the receptors, a very recent study by Li et al. [29] has used the approach of continuous-space diffusion on the basis of Monte Carlo simulation of the Langevin dynamics. Using photoactivated localization microscopy (PALM) technique, the spatial distribution of the submembranous PSD-95 binding obstacles was determined and the other reflecting transmembrane obstacles in the simulation space were distributed accordingly. The findings of their study strongly asserts the observations made in an earlier lattice model-based work by Sanatamaria et al. [31] from the same research group. The present study additionally raises a significant factor of self-crowding in shaping the receptors diffusion. Hence, what appears important is to show here how convergent is the lattice-diffusion approach and the proposed recursive algorithm to the self-crowded continuous-space diffusion.

Accordingly, the Monte-Carlo simulation of the Langevin dynamics of receptor diffusion is performed with steric-exclusion under the vibrant conditions of self-crowding. In this approach, the AMPARs are modelled as flat circular disks of exclusion radius 6*nm*. The centre of the disc is moved in random directions over a Δ*t* time-step of simulation as $\Delta x=\sqrt{2D\Delta t}\xi \;(t)$ and $\Delta y=\sqrt{2D\Delta t}\xi \;(t)$. Here, *ξ*(*t*) represents white Gaussian noise with mean zero and variance 1. The $\sqrt{2D\Delta t}$ defines the standard deviation of the random-sized steps taken independently in *x*− and *y*− directions over single time-steps of the simulation. *D* is the free diffusion coefficient of AMPARs in the postsynaptic membrane. While diffusion, it is taken care that the centres of any two discs should not be at a relative distance shorter than the double of the radius of the discs to implement steric-exclusion. This minimum distance between the centres of two discs represent collision between the incompressible hard discs. Further, the collisions are considered elastic. The number of these circular disks in a square simulation space of area 1*μm*^2^ is computed from the desired area fraction *a*_*DT*_ under investigation. The simulations begin with all the disks uniformly distributed across the simulation space with non-overlapping steric conditions. Three kinds of self-crowded conditions with sparse (*a*_*DT*_ = 0.0001), fairly dense (*a*_*DT*_ = 0.01 and 0.1) and extremely dense (*a*_*DT*_ = 0.6) presence of AMPARs are enquired. These three kinds of self-crowded conditions are chosen in accordance with the order of AMPAR density typically observed in the extra-synaptic membranes and the PSD, as mentioned above. The presence of any other obstacles is not considered.

**Fig 9:**
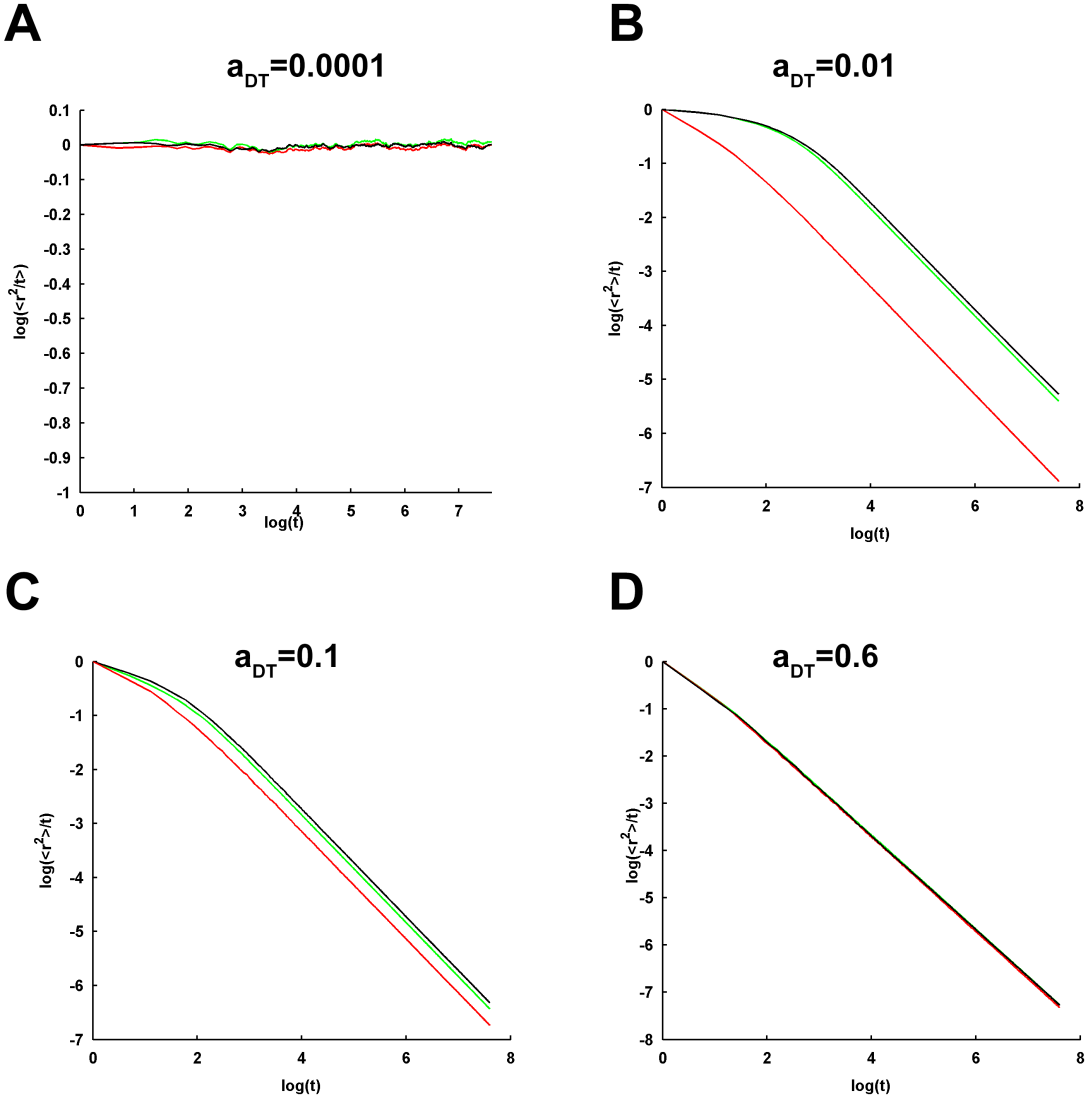
Normalized log-log profiles of the mean-squared displacements of diffusing receptors obtained from the continuous-space and lattice models of diffusion. The convergence of the proposed recursive algorithm for lattice model of self-crowded diffusion to the continuous-space Monte-Carlo simulation of Langevin dynamics is examined under the different self-crowding conditions, shown in (A) *a*_*DT*_ = 0.0001, (B) *a*_*DT*_ = 0.01, (C) *a*_*DT*_ = 0.1 and (D) *a*_*DT*_ = 0.6. Moreover, the efficacy of the recursive algorithm over its other possible counterpart neglecting recursion is also checked. The presence of any other reflecting or binding obstacles is not considered. The log-log profiles of the MSD obtained from recursive algorithm (shown in green) closely resemble that obtained from continuous-space diffusion (black) consistently across the different values of *a*_*DT*_. However, the profiles obtained from non-recursive algorithm (red) are observed to converge only for either very low or extremely high *a*_*DT*_. For other intermediate values of *a*_*DT*_, there lies considerable deviations from the outcomes of continuous-space diffusion scheme.

From the continuous-diffusion scheme, the log-log plots of the MSD are obtained by averaging over a sufficiently large size (700) of ensemble of the independent simulations. Based on this, the convergence of the newly-proposed lattice-based recursive algorithm for self-crowded diffusion to the continuous-space diffusion is checked and its efficacy over the other possibility without involving the recursion or repeated check of the labelled “DT-blocked tracers” in the same algorithm is also evaluated. Fig 9 demonstrates the overlaid normalized MSD plots obtained from the continuous-diffusion simulation, recursive lattice-based algorithm and the same algorithm without recursion under the different conditions of self-crowding. Interestingly, it is consistently observed that lattice-diffusion scheme with recursive algorithm is quantitatively sufficiently close to the MSD-profiles of the continuous-space diffusion in comparison to that without involving the recursive algorithm. For very low (*a*_*DT*_ = 0.0001) as well as very high (*a*_*DT*_ = 0.6) self-crowding density, it is quite apparent that the recursive algorithm and its absence are providing fairly identical convergence to the continuous-space model. Such convergence of the two lattice-diffusion schemes under very low density of tracers may be due to the lack of a substantial frequency of self-obstructing events. However, the same observed under very dense crowding of the tracers may arise from the fact that the fraction of false self-blocking events is negligible amidst the very frequent steric-collisions among the receptors, as most of the tracers are unable to diffuse under such strongly crowded condition. It is only for the intermediate densities (*a*_*DT*_ = 0.01 and 0.1) of the tracers that the distinction between the MSD profiles obtained from the schemes with and without the recursive algorithm is more conspicuous. It describes that majority of the obstructions observed on first-attempt through the computational sequence of hopping appears to be false and, as a result, the observed log-log plot appears steeper (i.e. more anomalous and obstructed) than that obtained from continuous-space schemes and recursive algorithm. However, when such obstructions are checked back in a recursive manner, it leads to their diffusion. Here, the behaviour of receptor diffusion in the absence of recursive algorithm is substantially deviated from that obtained from the continuous-space diffusion. More specifically, the deviation is higher for *a*_*DT*_ = 0.01 in comparison to *a*_*DT*_ = 0.1.

Seeing these remarkable consequences, an important question appears: why has the factor of self-crowding of the bulky AMPARs remain unappreciated till now when we already have a sufficiently large body of experimental data on the nature of AMPAR diffusion at excitatory synapses? Possibly, the reason to this ignorance does not entirely or essentially owe to the experimental studies. In fact, the in vivo sophisticated microscopic tracking of endogeneously-expressed AMPARs or less bulky genetically-engineered transmembrane probes at excitatory synapses and the resulting observations regarding their anomalous diffusion within PSD indeed involve all the several factors which are simultaneously present there under the real physiological conditions of the experiments [24–29]. However, the mechanistic deduction of the effects of these pertinent factors to the finer details is beyond the scope of any existing experimental techniques. At this point, the theoretical studies [29–33] using detailed models of the receptor diffusion in the presence of PSD crowd comes forth as the only but efficient option to dig deeper into the mechanisms. Certainly, these studies have so far led us to realize the impacts of obstruction and binding by the local crowd of transmembrane and submembrane scaffold proteins on receptor diffusion in the PSD. On the basis of these factors, the previous experimental data on the tracking of receptor diffusion has also been explained to a great extent. Therefore, the existing ignorance towards the possibility of an additional role of the self-crowding of receptors owes merely to the lack of consideration of the self-crowding in the earlier theoretical approaches.

Yet, the possible contributions of self-crowding could be unknowingly by-passed in the earlier theoretical approaches by appropriate parameter estimation within the framework of the previous models and it is a strong possibility that self-crowding remained a hidden variable in the process. For instance, even in the detailed study by Li et al. [29], simulations used to describe the monitored diffusion of genetically-engineered single- or double-pass transmembrane probes using FRAP as well as sptPALM techniques considered a substantial fraction of AMPARs endogenously-expressed in the cultured hippocampal neurons as the part of static crowd only. It must be noted that transmembrane crowds like AMPARs are sufficiently mobile and may impact differently from the other relatively static crowd on the probe diffusion. Nonetheless, this immediately draws attention to the fact that the experimental data on the receptor diffusion should also contain the elements of self-crowding, besides the earlier recognized factors. To throw light on this aspect, some of the previous MSD data on the AMPAR diffusion at excitatory synapses are examined on the basis of the observations acquired in the present study and is discussed in the following subsection.

### Elements of self-crowding in the previous experimental observations on AMPAR diffusion at excitatory synapses

As stated above, the commonly observed density of AMPARs in a typically-sized PSD would lead to an occupied area fraction ranging 0.08 − 0.4. The present observations suggest that receptor diffusion would be strongly anomalous at this level of area fraction due to self-crowding, regardless of the other transmembrane obstacles. This leads to a confusing situation where importance of transmembrane obstacles becomes obsolete, whereas the earlier studies have shown that the steric repulsion by the obstacles is an indispensable and critically essential factor behind AMPAR trapping and accumulation within the PSD. This contradiction arises because of the difference between the configuration of the diffusion system employed here and that of the system under natural condition. The diffusion system used here has a periodic boundary condition at its edges, leading to a homogenous condition of obstruction or binding applied on a diffusing entity throughout an infinite two-dimensional space. On the other hand, receptors diffusing within the PSD at excitatory synapses can easily escape the local crowded condition by entering into the extrasynaptic space and, hence, the natural diffusion system is an open system.

Therefore, the configuration of the present system mainly captures the diffusive behaviour of a receptor as long as it is diffusing within the PSD region and the density of the receptors is in a perfect or quasi- steady state. This leads to the speculation that self-crowding of AMPARs cannot itself hold the accumulated density of the receptors if the steric repulsion by the other obstacles are completely removed. Rather, steric obstructions by the relatively static density of other transmembrane proteins may provide the initial as well as maintaining driving force by reducing the mobility of the receptors within the PSD and self-crowding may later come into action as the density rises to a certain required level. In fact, this might be possible as increase in reflecting obstacle density leads to consistent decrease in the *D*_*eff*_ but a sudden increase in anomalousity occurs only beyond a certain very high CRO density. This speculation would have a remarkable impact on the required concentration of transmembrane obstacles predicted theoretically to effectuate anomalous confined diffusion of the receptors within PSD.

**Fig 10:**
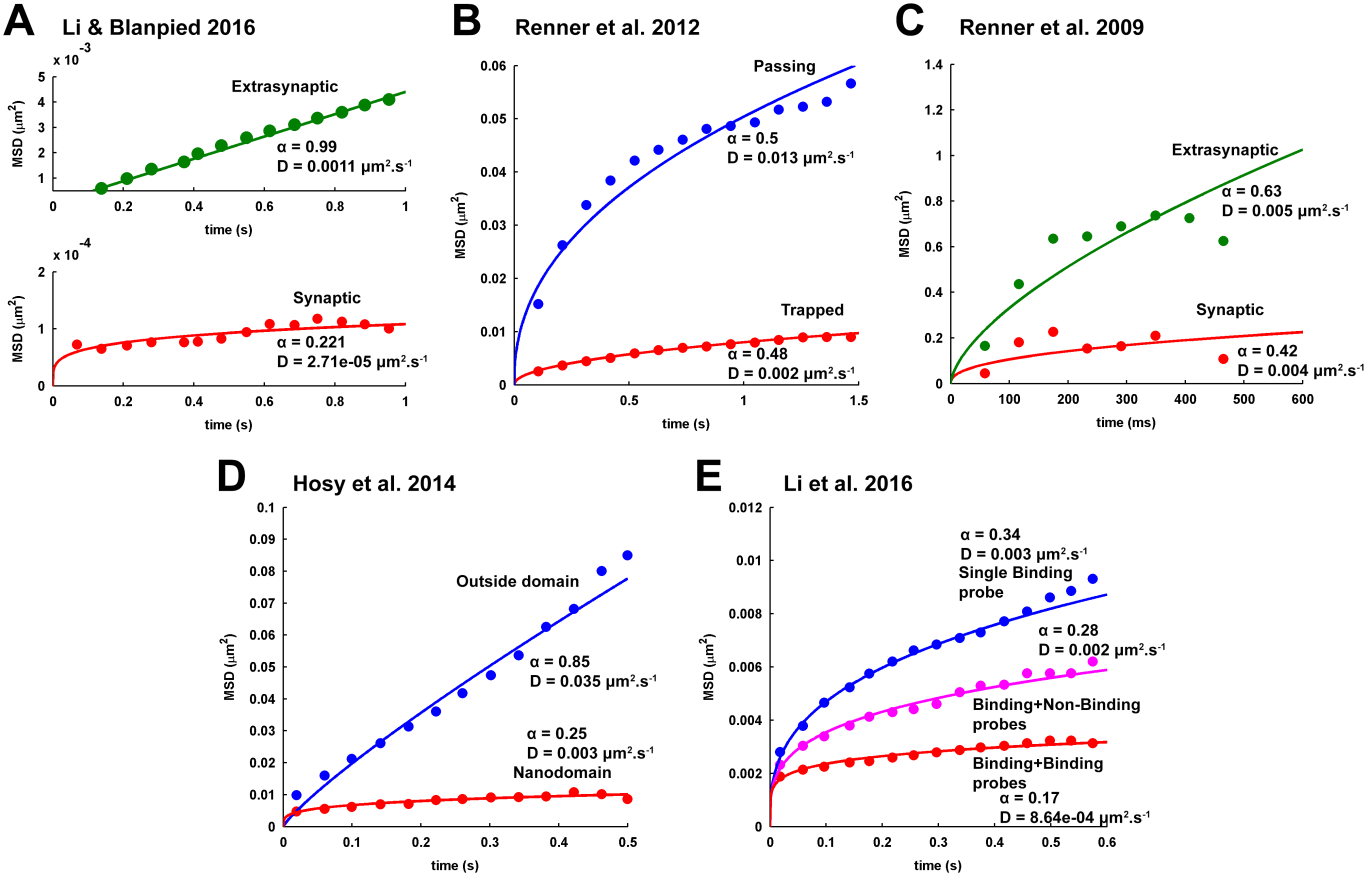
Analyzing some recent experimental data on the MSD of AMPAR diffusion at excitatory synapses for the anomalousity of receptor diffusion. The experimental data (A-E) on the nature of AMPAR diffusion, in terms of the temporal profiles of the MSD, obtained in the studies by Li and Blanpied (see ref. [76] in the main text), Renner et al. (ref. [24]), Renner et al. (ref. [77]), Hosy et al. (ref. [79]), and Li et al. (ref. [29]) using high-resolution microscopic techniques are shown in filled circles. Fitting of the standard relation between MSD and time for two-dimensional diffusion, given by 〈*r*^2^〉 = 4*Dt*^*α*^, to the data is shown with solid line. It yields the anomalous exponent *α* and the constant parameter *D* of the receptor diffusion. The estimated *α* of diffusion under different conditions are used here in the text to discuss the possible involvement of the self-crowding factor of AMPARs in the observed nature of their diffusion.

Fitting to the data on the diffusion of AMPARs in synaptic and extrasynaptic spaces acquired in the experimental study by Li and Blanpied [76] using single particle tracking and localization microscopy provides *α* = 0.22 and 0.99, respectively (Fig 10A). In the single-particle tracking experiment by Renner et al. [24] using quantum dots, two kinds of trajectories of the AMPARs diffusing in the PSD region were observed(Fig 10B). AMPARs, referred to as trapped, retained for longer durations within the PSD and exhibited strongly anomalous subdiffusion. The other population of AMPARs, referred to as passing, stayed for relatively shorter duration within the PSD but exhibited only a slightly lesser anomalous diffusion in comparison to the trapped receptors. Fitting to the MSD data of trapped and passing receptors provided *α* = 0.48 and 0.5, respectively. In a similar manner, through the fitting to the data on receptor diffusion within synaptic region obtained in the study by Renner et al. [77], the *α* comes out to be 0.42 (Fig 10C).

If the self-crowding factor is not considered, such high anomalousity of receptor diffusion could be possible only at an obstacle density (*a*_*CRO*_) between 0.4 and 0.44. Even, the theoretical study by Santamaria et al. [31] predicts a similar range of obstacle concentration (0.4 − 0.46) for achieving such low anomalous exponent of AMPAR diffusion. However, introduction of self-crowding can bring similar high levels of anomalousity even at lower levels of obstacle concentration (see Fig 4). Imagining that expression of transmembrane obstacles at a density lesser than the theoretically-predicted value would cause sharp loss in accumulated AMPAR density seems very strict and unrealistic for the natural scenario. In fact, self-crowding may provide a certain degree of flexibility to this aspect of synaptic homeostasis. This feature can be tested through an experiment where the nature of AMPAR diffusion and receptor accumulation within the PSD is examined under different densities of transmembrane obstacles. If a significantly anomalous receptor diffusion is observed at an obstacle density ammounting to occupied area fraction lesser than the abovementioned, it would be a strong evidence for the speculation drawn here for the self-crowding of the AMPARs.

Further, the average density of PSD-95 scaffold proteins in the PSD of an excitatory synapse is known to be 3000*μm*^−2^ [78]. The radial size of a PSD-95 protein is estimated to be almost 2.5*nm* [35], which results into a lateral span of 19.64 × 10^−6^*μm*^2^. Assuming a homogenous distribution of the PSD-95, the area fraction of the PSD occupied submembranously by the total PSD-95 proteins would amount to 0.059. Knowing that AMPARs are present at very high density within the PSD [74, 75], the present study suggests that binding in the presence of such low area fraction of PSD-95 would cause an insignificant effect on the anomalousity of receptor diffusion, unless the AMPAR-PSD-95 binding affinity is extremely high throughout the binding sites. Interestingly, this has also been noted in the earlier experimental study by Li and Blanpeid [76], stating that whole-synapse PSD-95 density would have inconsiderable impact on the diffusion of transmembrane proteins.

However, the experimental estimation of PSD-95 distribution demonstrates that, rather than homogeneously distributed, these proteins are enriched in smaller subregions or nanodomains within the PSD [52]. Accordingly, their local density and occupied area-fractions within these nanodomains may acquire considerably large magnitudes, such that even moderate binding affinity may appear effective in reducing the anomalousity of receptor diffusion at high receptor density within the nanodomains. Therefore, such PSD-95 distribution appears as a physiological strategy to enhance the effectiveness of binding on the AMPAR diffusion and exchange with the perisynaptic space.

Nonetheless, AMPARs have also been observed to accumulate at higher density within the nanodomains [52, 79]. The present observations suggest that, given PSD-95 density and AMPAR-binding affinity, the extent to which the anomalousity arising from the self-crowding would decrease further depends on the local receptor density within these domains. As the area fraction occupied by the higher density of AMPARs within the much smaller (∼ 80*nm*, [79]) PSD-95-rich domains would be very large, receptor diffusion within scaffold-rich domains would retain significant anomalousity, despite the ameliorating influence of binding on the anomalousity. In fact, experimental observations using super-resolution microscopy in the study by Hosy et al. [79] on the nature of AMPAR diffusion within these nanodomains and outside (Fig 10D) indicate that the AMPAR diffusion within these PSD-95-rich nanodomains indeed exhibit strongly anomalous diffusion. Fitting to their data on receptor diffusion within the nanodomains and in peripheral region provides *α* = 0.25 and 0.85, respectively. It must be noted that nanodomains do contain transmembrane obstacles [5], for instance the adhesion proteins LRRTM2 [80]. However, the AMPARs are present at an extraordinarily high density in these nanodomains and self-crowding appears to be a prominent factor underlying the intense anomalousity in receptor diffusion observed earlier [79].

In a recent study by Li et al. [29] using genetically-engineered single-pass transmembrane probes, it has been observed that rapalog-mediated cross-linked binding probes with intracellular PDZ-binding segments exhibit more anomalous and confined diffusing than the single binding probes (Fig 10E). Fitting to the data provides *α* = 0.34, 0.28 and 0.17 for the single probes, binding-non-binding cross-linked probes and binding-binding cross-linked probes, respectively. In a straightforward manner, it appears that enhanced binding with multiple PDZ-domains enhances anomalousity of receptor diffusion. However, it can be also be possible that increase in binding lowers the receptor mobility and causes accumulation of larger number of AMPARs in the local area. This may lead to increased self-crowding in a feedback manner and fuels stronger anomalousity to the receptor diffusion trapping more receptors.

Nonetheless, the major practical limitation towards drawing a definitive conclusion is that the earlier experimental studies haven’t taken into account the density of AMPARs at the PSD while tracking their diffusive properties and, in the absence of such mentions, it is difficult to establish the connection between the exact contribution of self-crowding of AMPARs to their overall nature of diffusion within the PSD. Besides this, the present study is also in its preliminary state and contains some important limitations when it comes to closely imitate the true biological condition. Given a population of AMPARs, the receptors may be in different states which may affect the strength of binding to the scaffold proteins, such as association with different kinds of auxiliary transmembrane proteins [39, 45] or no association at all [56, 57], glutamate-bound desensitized state of the receptor [81], differences in the cytoplasmic domains of receptor subtypes [56, 57] etc. The phosphorylation state of the auxiliary transmembrane protein, such as Stargazin [82] or that of the scaffold proteins, such as PSD-95 [83], also significantly affects binding. Together, these factors may lead to heterogeneous distribution of binding strength across the PSD. Further, the spatial distribution of the various scaffold proteins is inhomogeneous and smaller sub-regions within the PSD are found to have higher density of these proteins [52, 53]. In a similar manner, various transmembrane proteins such as N-cadherin, have specific spatial distributions [29, 35] rather than a perfectly homogeneous distribution in the PSD assumed here. Further, the presence of extracellular matrix in the synaptic cleft has also been observed to affect the lateral diffusion and accumulation of AMPARs in the PSD [84].

However, despite these limitations at the level of finer details, the present investigation indeed serves as an initial step towards gaining insight into the aspect of self-crowding of AMPARs, and similar other mobile bulky transmembrane proteins, and its effect on lateral diffusion in the postsynaptic membrane as well as in the specialized crowded PSD region. These features may serve as the conceptual nut-bolts for understanding the behaviour of more detailed models capturing the true irregular topology of synaptic PSD and the natural spatial distributions of the crowding and binding elements. Further investigations may involve the effects of self-crowding on the dynamics of AMPAR trapping and accumulation under the conditions of house-keeping maintenance of synaptic strength and evoked synaptic plasticity.

## Supporting information

S1 Video Sample trajectories of self-crowded diffusion. The temporal evolution of the *x*− and *y*− positions of a diffusing tracer under the different self-crowded conditions are shown for 1*s* duration of the simulation of lattice diffusion model with the recursive algorithm. The positions are recorded at every 1*ms*. A control condition of completely-unobstructed free-diffusion of a tracer is also shown for comparison. The simulation does not include any kind of reflecting or binding obstacles. *a*_*DT*_ represents the area-fraction of the lattice occupied by the tracers.
(AVI)
